# COVID-19 length of hospital stay: a systematic review and data synthesis

**DOI:** 10.1186/s12916-020-01726-3

**Published:** 2020-09-03

**Authors:** Eleanor M. Rees, Emily S. Nightingale, Yalda Jafari, Naomi R. Waterlow, Samuel Clifford, Carl A. B. Pearson, CMMID Working Group, Thibaut Jombart, Simon R. Procter, Gwenan M. Knight

**Affiliations:** 1 Department of Infectious Disease Epidemiology, London School of Hygiene and Tropical Medicine, Keppel Street, London, UK; 2 Department of Global Health and Development, London School of Hygiene and Tropical Medicine, Keppel Street, London, UK; 3 South African DSI-NRF Centre of Excellence in Epidemiological Modelling and Analysis (SACEMA), Stellenbosch University, Stellenbosch, Republic of South Africa; 4 UK Public Health Rapid Support Team, London, UK; 5 MRC Centre for Global Infectious Disease Analysis, Department of Infectious Disease Epidemiology, School of Public Health, Imperial College, London, UK

**Keywords:** Hospitalisation, ICU capacity, COVID-19, SARS-CoV-2, Bed demand, Length of stay

## Abstract

**Background:**

The COVID-19 pandemic has placed an unprecedented strain on health systems, with rapidly increasing demand for healthcare in hospitals and intensive care units (ICUs) worldwide. As the pandemic escalates, determining the resulting needs for healthcare resources (beds, staff, equipment) has become a key priority for many countries. Projecting future demand requires estimates of how long patients with COVID-19 need different levels of hospital care.

**Methods:**

We performed a systematic review of early evidence on length of stay (LoS) of patients with COVID-19 in hospital and in ICU. We subsequently developed a method to generate LoS distributions which combines summary statistics reported in multiple studies, accounting for differences in sample sizes. Applying this approach, we provide distributions for total hospital and ICU LoS from studies in China and elsewhere, for use by the community.

**Results:**

We identified 52 studies, the majority from China (46/52). Median hospital LoS ranged from 4 to 53 days within China, and 4 to 21 days outside of China, across 45 studies. ICU LoS was reported by eight studies—four each within and outside China—with median values ranging from 6 to 12 and 4 to 19 days, respectively. Our summary distributions have a median hospital LoS of 14 (IQR 10–19) days for China, compared with 5 (IQR 3–9) days outside of China. For ICU, the summary distributions are more similar (median (IQR) of 8 (5–13) days for China and 7 (4–11) days outside of China). There was a visible difference by discharge status, with patients who were discharged alive having longer LoS than those who died during their admission, but no trend associated with study date.

**Conclusion:**

Patients with COVID-19 in China appeared to remain in hospital for longer than elsewhere. This may be explained by differences in criteria for admission and discharge between countries, and different timing within the pandemic. In the absence of local data, the combined summary LoS distributions provided here can be used to model bed demands for contingency planning and then updated, with the novel method presented here, as more studies with aggregated statistics emerge outside China.

## Background

As of April 28, 2020, there have been over 3 million confirmed cases of COVID-19 and more than 200,000 deaths across 185 countries and territories [1]. Health systems are challenged by the influx of patients as SARS-CoV-2, the pathogen causing COVID-19, has spread throughout the world since its emergence in late December 2019 [2–6]. The risks of healthcare services being overwhelmed were most dramatically illustrated in Italy, where a rapid increase of COVID-19 cases needing hospitalisation pushed a well-equipped health system of 3.2 hospital beds per 1000 people to breaking point [7]. This raises serious concerns over the potential impact on more resource-constrained health systems in low- and middle-income countries (LMICs) as epidemics begin to expand across Africa and South America.

Understanding and predicting hospital bed demand (as well as associated staff or equipment requirements) provide crucial evidence for decision-making and contingency planning (7, 8). Predicting demand for hospital services requires an estimate of the number of patients requiring hospitalisation and an estimate of how long each person will require hospital care. It is possible to model the rate of hospitalisation in many settings based on estimated epidemic curves. However, estimating length of stay (LoS) in hospitals requires observation of individual patient pathways.

COVID-19 presents at varying levels of severity. Hospital care can vary from general ward-based care to high dependency units with oxygen support to intensive care where patients may be intubated for mechanical ventilation [8–10]. The LoS is likely to depend on the level of care required, as well as the geographic setting due to varying COVID-19 care guidelines. For example, some hospitals in China were initially used as isolation settings [11, 12]. As knowledge of effective treatments changes, the pathways, staff, beds, and equipment required are also likely to affect the duration and level of care needed. Moreover, patient characteristics—such as age and comorbidities—impact disease severity [8, 12–14] and are likely to influence LoS. If differences are significant, then capacity planning may need to account for these characteristics to provide accurate predictions of the number of beds required at each level of care. Modelling studies predicting bed occupancy published so far have broadly relied on very few sources of information for LoS estimates, which were often derived from very different settings [15–22]. Estimates for LoS can be obtained from a variety of studies, but are often an incidental result rather than a study’s primary outcome, and typically, only summary statistics are reported. In general, LoS distributions are right-skewed due to a minority of patients with long hospital stays and are often modelled using gamma, log-normal, or Weibull distributions [23] (although log-normal is less preferred due to its heavier tails). A particular challenge is how to synthesise appropriate LoS distributions from a range of relevant sources in similar settings, capturing the variation both within and between them. Incorporating the uncertainty and stochasticity in parameters using a distribution, rather than fixed point estimates (such as the mean over all studies), allows for more realistic model predictions.

We performed a systematic review to identify the current evidence on LoS for COVID-19 patients worldwide. We also present a method for generating LoS summary distributions by combining information from different summary statistics (mean and medians) reported in multiple studies, and accounting for differences in sample sizes. This aims to include all the variation between studies, to obtain a distribution that covers all plausible LoSs. Although similar in the sense of synthesising multiple sources, this is unlike a classic meta-analysis which aims to get a more precise estimate of a quantity assumed as being a fixed point value. In doing this work, we aim to inform the efforts of modellers and policy makers to better anticipate healthcare needs during the evolving COVID-19 pandemic.

## Methods

### Search strategy

This study was conducted following the Preferred Reporting Items for Systematic Reviews (PRISMA) guidelines (9). We searched the bibliographic databases Embase and Medline, as well as the online pre-print archive medRxiv. The latter was expected to be an important source due to the current rapid development of this field; hence, the fully published literature would capture only a small proportion of the available information. We included articles published up to 12 April 2020 that reported a LoS for COVID-19 patients admitted to hospital. To ensure all relevant papers were captured, we examined the title, abstract, and keywords of known studies reporting LoS to identify relevant search terms. Our search combined the concepts of COVID-19 (coronavirus, COVID-19, 2019-nCov, and SARS-CoV-2) with search terms related to duration of hospital stay (length of stay, admission duration, admission length, hospital*). The search terms for hospital stay length were kept broad to capture studies that report LoS as a secondary outcome. The full search terms for Embase, Medline, and medRxiv are presented in the supplementary materials. In addition to our systematic searches, we also checked situation reports from the following organisations to see if they reported LoS estimates: UK Intensive Care National Audit and Research Centre (ICNARC), International Severe Acute Respiratory and Emerging Infection Consortium (ISARIC), World Health Organization (WHO), the US Centers for Disease Control and Prevention (CDC), and China CDC and European CDC (ECDC).

### Inclusion and exclusion criteria

Inclusion criteria are as follows:

1. Studies that reported LoS in hospital for individuals who were admitted for confirmed COVID-19, or suspected COVID-19 which was later confirmed
2. Published (either as a pre-print or publication) between 1 January 2020 and 12 April 2020

Exclusion criteria are as follows:

1. Studies were excluded if LoS was reported for individuals only admitted to hospital for a reason other than confirmed or suspected COVID-19
2. Studies where the LoS endpoint was not death or discharge or continuing stay, for example, transfer to another hospital
3. Studies which stated that hospitalisation was used as a form of isolation
4. Studies not published in English
5. Review articles

### Screening

**Fig. 1:**
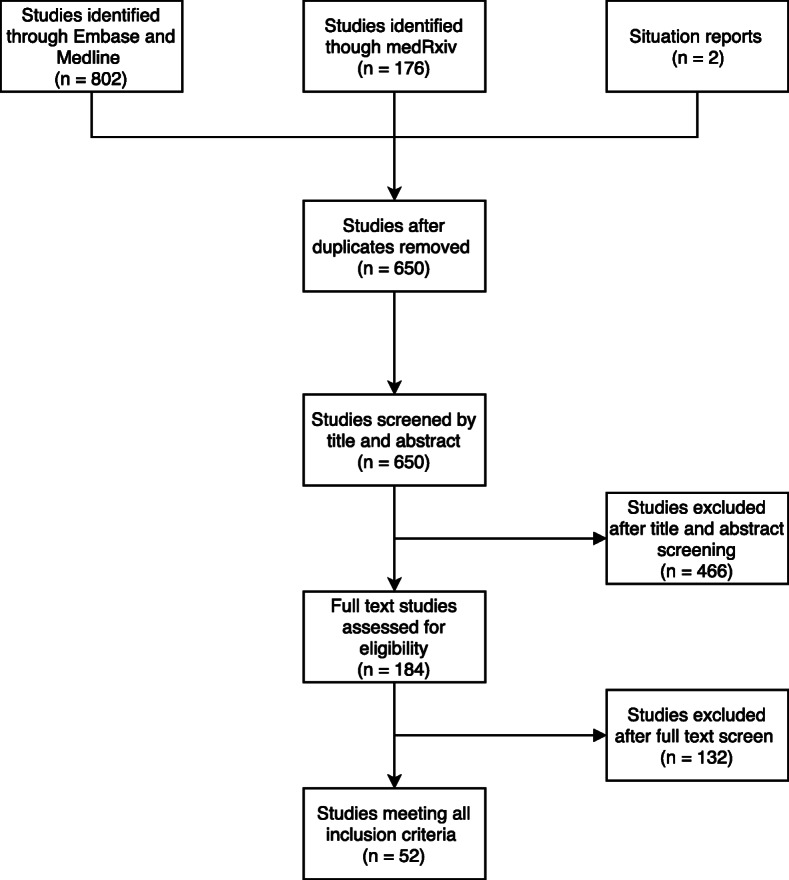
PRISMA diagram showing the results of the screening process used to identify included studies (*n*=52)

The screening process is summarised in Fig. 1. All titles and abstracts were screened independently by two reviewers (EMR and SRP). Subsequently, abstracts and full texts of potentially relevant papers were independently reviewed by two reviewers (EMR and YJ).

### Data extraction and analysis

The data that was extracted from each study is presented in Supplementary Table A. Data extraction was performed by EMR, YJ, and ESN and then verified by a second member of the study team. Study characteristics (such as study dates, study population, and study design) were recorded from each study, including information on the LoS sample estimate for both total hospital LoS and intensive care unit (ICU) LoS, as well as sample size, discharge status, and completeness of follow-up. If multiple LoS estimates were reported for different study populations, these were all recorded (for example, LoS reported by disease severity, comorbidities, and treatment groups). One study specified non-ICU LoS, and this was grouped with total LoS estimates; ICU LoS was reported separately. Average patient age and sex distribution (% male) were summarised across all studies by weighted mean and standard deviation (mean (SD)), according to study sample size. Where possible, LoS estimates were recorded as median and interquartile range (IQR). Otherwise, mean and SD, or in some cases, only a point estimate was provided.

An assessment of bias was considered in these studies. Reported LoS in the majority of studies was not the primary outcome; therefore, a formal quality appraisal was not considered appropriate. Instead, we considered biases which may directly affect reported LoS. Studies were assessed as to whether follow-up had been completed for all included patients, such that an outcome of discharge or death had been observed for all and none remained in hospital at the end of the study. If this was not the case, the final summary of LoS may be biassed by including only shorter stays which were resolved within the time frame. As reported above, summaries of patient characteristics and comorbidities were recorded for each study where possible; this allowed later comparison of estimates based on features of the study population and provided some additional context with which to interpret the LoS estimates. Finally, the risk of bias related to potentially overlapping populations was also considered. Studies from the same hospital over the same time period were identified, and the study with largest sample size was selected from that group. A list of these studies is presented in Supplementary table B. LoS was then summarised including only these selected studies from the overlapping groups, along with the other non-overlapping studies. This estimate was then compared to the estimates based on all studies.

### Estimating LoS distributions

Overall summary distributions were created for total hospitalisation LoS and for ICU LoS. Unlike a meta-analysis, the assumption here was not that each study had estimated an average LoS with some error, but that each study had captured a portion of the overall LoS distribution across a general population. Each of the studies provides us with a “sample” which we use to generate a better idea of the “true” underlying distribution. Our aim was to therefore incorporate all the variation across studies in order to obtain a distribution covering all plausible LoS values. We included studies in the estimation of these summary distributions if they reported both the sample size along with either the median and IQR or the mean and standard deviation. If no measure of variation was provided (either IQR or standard deviation), the point estimates were included in figures but excluded from these summary results.

Weibull distributions were fitted to the summary data from each study, using Nelder-Mead optimisation (implemented in the *stats* package in R [24]) for those reporting medians and IQRs. Specifically, the shape and scale parameters were varied in order to minimise the squared distance between the distribution and study quantiles. Where estimates were presented as a mean, *x*, and standard deviation, *s*, the distribution was fitted by moment-matching using the *mixdist* package [25]. The same approach was also tested using gamma distributions, but Weibull was marginally preferred with respect to total squared error in the fitted quantiles. These distributions were then discretised using the *distcrete* package in R [26]. A total of 100,000 samples were then drawn from each of these distributions, with weighting according to their sample size. Specifically, the study distributions were first sampled according to a multinomial distribution defined by the studies’ relative sample sizes, and LoS was then sampled from each of these sampled distributions. Due to potential important differences in the characteristics of each study population, it may not be appropriate to weight entirely on sample size without considering how representative the cohort is of the general population. Therefore, as a sensitivity analysis, we performed the same analysis without weighting in order to understand how much this influences the distribution. In some cases, studies reported LoS according to some stratification and not over the whole study population. Here, we applied the same method to summarise across the strata and obtain an estimated median and IQR across the whole population, validating the approach using examples where the overall summary statistics were also provided.

All analyses were performed using R version 3.6.3 (29 February 2020).

## Results

### Study characteristics

The results of our screening process are summarised in Fig. 1. After removing duplicates, we found a total of 650 potentially eligible studies of which 52 studies met all the inclusion criteria. These included 32 peer-reviewed articles from the academic literature, 18 pre-print articles, and 2 reports from other sources ([27] and [28]). Several studies reported LoS by specific patient subgroups, according to disease severity, comorbidities (kidney injury, liver injury, hypertension, and cardiac injury), experimental treatments (heparin, lopinavir-ritonavir), and pregnancy status. A complete description of all reported LoS estimates is provided in Supplementary Table B. The key characteristics of the included studies are summarised in Table 1.

**Table 1:** Summary study characteristics of included studies (*n*=52). A total of 46 studies were identified from China, and 6 studies identified outside of China

Author	Publication date	Status	Study dates	Country	Province/ region	Study population	Study design	Sample size LOS	Sex (% male)	Age	Follow-up completed	Notes
Bhatraju et al. [34]	30 March 2020	Published	24 February 2020–09 March 2020	USA	Seattle, WA	Patients with laboratory-confirmed COVID-19 infection who were admitted to nine hospital ICUs in the Seattle region	Cohort	24	63	64 ± 18**	No	Not all patients in this study were discharged. Once discharged from ICU, several patients were transferred to ward.
Cai et al. [60]	02 April 2020	Published	11 January 2020–06 February 2020	China	Shenzhen, Guangdong	Patients with confirmed COVID-19 admitted to the Third People’s Hospital of Shenzhen	Cohort	298	48.66	47.5 (33–61)*	No	Patients remained hospitalised at the end of the study. It was unclear whether LOS was reported for only dead/discharged patients or also included still-hospitalised patients.
Cao et al. [61]	18 March 2020	Published	18 January 2020–03 February 2020	China	Wuhan, Hubei	Laboratory-confirmed COVID-19 patients admitted to Jin Yin-Tan Hospital	RCT	199	60.3	58 (49–68)*	No	Patients still hospitalised at the end of the study. Not clear on the number of deaths.
Cao et al. [62]	02 April 2020	Published	03 January 2020–15 February 2020	China	Wuhan, Hubei	All lab-confirmed patients with COVID-19 admitted to Wuhan University Zhongnan Hospital	Cohort	102	52	54 (37–67)*	Yes	
Chen et al. [63]	11 March 2020	Published	20 January 2020–25 February 2020	China	Shanghai	Recruited patients admitted to Shanghai Public Health Clinical Center (SPHCC), diagnosed with COVID-19 according to Chinese national guideline for COVID-19 diagnosis and treatment, as well as the World Health Organization interim guidance.	Cohort	215	50.6	51 (36–64)*	No	Not all patients in this study were discharged.
Chen et al. [64]	26 March 2020	Published	13 January 2020–28 February 2020	China	Wuhan, Hubei	All COVID-19 confirmed patients admitted to Tongji Hospital	Cohort	274	62.4	62.0 (44.0–70.0)*	No	Patients still hospitalised at the end of the study.
Chen et al. [65]	06 March 2020	Published	23 January 2020–20 February 2020	China	Hunan	All patients with confirmed COVID-19 admitted to the First Hospital of Changsha and Loudi Central Hospital	Cohort	159	49.8	46 (34–59)*	No	Two estimates of LOS were provided in this study: one estimate for discharged patients only and one estimate for all patients (including still-hospitalised). The estimate which included still-hospitalised patients was excluded.
Cheng et al. [66]	20 March 2020	Published	28 January 2020–17 February 2020	China	Wuhan, Hubei	All COVID-19 patients admitted to Tongji Hospital	Cohort	113	52.4	63 (50–71)*	No	Patients still hospitalised at the end of the study. LOS was only reported for deaths.
Deng et al. [67]	20 March 2020	Published	01 January 2020–21 February 2020	China	Wuhan, Hubei	Recovered and dead patients admitted two tertiary hospitals in Wuhan (Hankou and Caidian branch of Tongji Hospital, Tongji Medical College, Huazhong University of Science & Technology, and Hankou branch of Central Hospital of Wuhan)	Cohort	225	55.1	21–94***	Yes	Only enrolled dead and discharged patients within the study, so data is still censored.
Ding et al. [68]	26 March 2020	Published		China	Wuhan, Hubei	Patients infected with both influenza and COVID-19 admitted to Tongji Hospital	Cohort	5	40	39–66***	Yes	
Du et al. [69]	03 April 2020	Published	09 January 2020–15 February 2020	China	Wuhan, Hubei	Severe patients with confirmed COVID-19 admitted to Hannan Hospital and Wuhan Union Hospital	Cohort	85	72.9	65.8 ± 14.2**	Yes	LOS was only reported for deaths.
Fan et al. [70]	05 March 2020	Published	20 January 2020–19 February 2020	China	Shanghai	All confirmed COVID-19 cases in Shanghai Public Health Clinical Center	Cohort	93	49.3	50.5 (36–64)*	No	Patients still hospitalised at the end of the study.
Graselli et al. [32]	06 April 2020	Published	20 February 2020–25 March 2020	Italy	Lombardy	All patients with laboratory-confirmed COVID-19, referred to Ospedale Maggiore Policlinico, and subsequently admitted to one of the ICUs amongst 72 hospitals in the network	Cohort	1591	82	63 (56–70)*	No	Patients still hospitalised at the end of the study.
Guan et al. [36]	28 February 2020	Published	11 December 2019–31 January 2020	China	National	Hospitalised lab-confirmed cases of COVID-19 from 552 hospitals in 30 provinces in China	Cohort	1099	58.1	47.0 (35.0–58.0)*	No	1029/1099 patients were still hospitalised.
ICNARC [28]	16 April 2020	Other	16 April 2020	UK	National	Patients critically ill with confirmed COVID-19 reported to ICNARC up to 16 April 2020 from critical care units participating in the Case Mix Programme. This is a national audit that is being continually updated.	Cross-sectional	2936	72.1	60 (52–68)*	No	Patients still hospitalised at the end of the study.
ISARIC [27]	08 April 2020	Other	25 March 2020	Worldwide (25 countries)		Hospitalised patients with confirmed or suspected COVID-19 submitted electronically by participating sites to the ISARIC database.	Cohort	3316	0.5	71*	No	Patients still hospitalised at the end of the study. Age and sex are only available for all patients. Majority of data is from the UK (82%). LOS estimates were only provided for discharges and deaths.
Liu et al. [71]	13 March 2020	Pre-print	16 January 2020–15 February 2020	China	Wuhan, Hubei	Confirmed cases of COVID-19 medical staff admitted to Union Hospital	Cohort	64	36	35 (29–43)*	No	Patients still hospitalised at the end of the study.
Liu et al. [37]	12 March 2020	Published	22 January 2020–11 February 2020	China	Hangzhou, Zhejiang	Patients with confirmed COVID-19, admitted to Xixi hospital	Cohort	10	40	42 (34–50)*	No	Patients still hospitalised at the end of the study (3 transferred to ICU).
Liu et al. [72]	23 February 2020	Pre-print	20 January 2020–03 February 2020	China	Chongqing	Hospitalised patients with confirmed COVID-19 at Chongqing University Three Gorges Hospital	Cohort	51	62.7	45 (34–51)*	Yes	
Mo et al. [73]	16 March 2020	Published	01 January 2020–05 February 2020	China	Wuhan, Hubei	All confirmed COVID-19 patients admitted to Zhongnan Hospital	Cohort	22	Missing	Missing	No	LOS is reported for both deaths and discharges; however, sample size was only reported for deaths, so the LOS for discharges was excluded. Age and sex of the population were not provided.
Pan et al. [74]	13 February 2020	Published	12 January 2020–06 February 2020	China	Wuhan, Hubei	Patients with RT-PCR confirmed COVID-19 infection presenting at Union Hospital, Tongji Medical College. Patients with severe respiratory distress and/or oxygen requirement at any time during the disease course were excluded.	Cohort	21	29	40 ± 9**	Yes	Severe cases were excluded from this study.
Petrilli et al. [35]	11 April 2020	Pre-print	01 March 2020–07 April 2020	USA	New York, NY	All patients with laboratory-confirmed COVID-19 treated at an academic health system in New York City (NYU Langone Health)	Cross-sectional	1273	50.5	52 (36–65)*	No	In the critical illness, some patients still hospitalised at the end of the study. In the non-critical, all patients discharged (no deaths).
Qi et al. [75]	03 March 2020	Pre-print	19 January 2020–16 February 2020	China	Chongqing	Laboratory-confirmed COVID-19 patients admitted to 3 designated hospitals (Qianjiang Central Hospital of Chongqing, Chongqing Three Gorges Central Hospital, and Chongqing Public Health Medical Center) in Chongqing provincial municipality	Cohort	164	55.8	48 (35–65)*	No	Patients still hospitalised at the end of the study. Not clear whether they have included patients still admitted to hospital or limited to only those that have been dead or discharged within their LOS estimate
Qui et al. [29]	25 March 2020	Published	17 January 2020–01 March 2020	China	Wenzhou, Zhejiang	Paediatric patients (aged 0–16 years) with confirmed COVID-19 from electronic medical records in three hospitals in Zhejiang, China (Ningbo Women and Children’s Hospital, The Third Affiliated Hospital of Wenzhou Medical University, and Wenzhou Central Hospital of Wenzhou)	Cohort	36	63.9	8 ·3 ± 3 ·5**	Yes	
Shen et al. [45]	27 March 2020	Published	20 January 2020–25 March 2020	China	Shenzhen, Guangdong	Laboratory-confirmed COVID-19 and acute respiratory distress syndrome (ARDS) who were admitted to Shenzhen Third People’s Hospital who met the following criteria: severe pneumonia with rapid progression and continuously high viral load despite antiviral treatment, PAO2/FIO2 <300, and mechanical ventilation.	Cohort	5	60	36–73***	No	Patients still hospitalised at the end of the study.
Shi et al. [30]	10 March 2020	Published	19 January 2020–15 February 2020	China	Shanghai	Children admitted to outpatient and emergency department with confirmed COVID-19 to Children’s Hospital of Fudan University, Shanghai	Cohort	10	50	6.0 ± 4.2**	No	Only 4 patients had been discharged when LOS was calculated.
Shi et al. [44]	07 April 2020	Pre-print	01 February 2020–15 March 2020	China	Wuhan, Hubei	Patients with COVID-19 (severe clinical classification) treated at the Union Hospital, Tongji Medical College, Huazhong University of Science and Technology	Cohort	42			Yes	No critical cases
Shi et al. [42]	25 March 2020	Published	20 January 2020–15 February 2020	China	Wuhan, Hubei	Patients with laboratory-confirmed COVID-19 admitted to Renmin Hospital	Cohort	49	53.7	64 (21–95)*	No	Only report LOS for those who have died: 77% remained in hospital
Shi et al. [43]	24 February 2020	Published	20 December 2019–08 February 2020	China	Wuhan, Hubei	Patients with confirmed COVID-19 admitted to Wuhan Jinyintan Hospital or Union Hospital of Tongji Medical College	Cohort	29	Missing	Missing	No	Patients still hospitalised at the end of the study.
Spiteri et al. [33]	05 March 2020	Published	24 January 2020–21 February 2020	EU/EEA	EU/EEA	European surveillance data of confirmed COVID-19 patients	Cross-sectional	17	Missing	Missing	No	Patients still hospitalised at the end of the study.
Tang et al. [76]	26 March 2020	Published	24 December 2019–07 February 2020	China	Wuhan, Hubei	Patients with COVID-19-induced ARDS admitted to the Department of Pulmonary and Critical Care at Wuhan Pulmonary Hospital in Hubei Province of China	Case control	73	61.6	67 (57–72)*	No	Not clear whether LOS included patients remaining in hospital, or if they were excluded.
Tian et al. [77]	23 March 2020	Pre-print	Not stated	China	Liaocheng, Shandong	Not stated	Cohort	37	45.9	44.3 ± 1.67**	No	Not clear what the study population is; location and hospital have been assumed, and study dates have not been provided. Patients still hospitalised at the end of the study.
Tian et al. [78]	07 April 2020	Pre-print	21 January 2020–05 March 2020	China	Jilin	Three designated tertiary hospitals in Jilin province including the First Hospital of Jilin University (*n*=3), Changchun Infectious Disease Hospital (*n*=42), and Siping Infectious Disease Hospital (*n*=14)	Cohort	59	57.6	41 (29–52)**	Yes	Dates are different in abstract and main text.
Wang et al. [39]	20 March 2020	Published	01 January 2020–05 March 2020	China	Wuhan, Hubei	All confirmed cases of COVID-19 over 60 years old admitted to Renmin Hospital of Wuhan University in Wuhan, China.	Cohort	339	49	69 (65–76)*	No	LOS was provided for survivors; however, not all survivors had been discharged (91/274), and so this estimate includes still-hospitalised patients.
Wang et al. [38]	10 April 2020	Pre-print	16 January 2020–04 March 2020	China	Sichuan	Laboratory-confirmed cases of COVID-19, which were reported to the Sichuan Center for Disease Control and Prevention (CDC) through the National Notifiable Diseases Reporting System (NNDRS).	Cross-sectional	538	53	Missing	No	Not clear whether patients still-hospitalised were included in the LOS estimate, we have assumed that still-hospitalised patients were included.
Wu et al. [55]	29 February 2020	Pre-print	25 December 2019–11 February 2020	China	Wuhan, Hubei	COVID-19 patients admitted to Wuhan Jinyintan Hospital	Cohort	188	63.3	51.9 ± 14.26**	Yes	
Wu et al. [79]	29 February 2020	Published	22 January 2020–14 February 2020	China	Jiangsu	All admitted patients for COVID-19 to First People’s Hospital of Yancheng City, the Second People’s Hospital of Yancheng City, and the Fifth People’s Hospital of Wuxi.	Cohort	21	48.8	46.10 ± 15.42**	No	80 patients but LOS only reported for those discharged.
Wu et al. [80]	13 March 2020	Published	25 December 2019–13 February 2020	China	Wuhan, Hubei	Confirmed COVID-19 pneumonia admitted to Wuhan Jinyintan Hospital	Cohort	144	63.7	51 (43–60)*	No	Unclear whether LOS included those who died. State that some of their cases have previously been described in other studies. Age and sex are regarding total population (including those remaining in hospital).
Wu et al. [81]	09 April 2020	Pre-print	26 February 2020	China	Shanghai	Adult COVID-19 patients admitted to Shanghai Public Health Clinical Center.	Cohort	175	47	50* (16–85)***	Yes	Only enrolled discharged and mild patients. No start date for the study provided.
Xia et al. [31]	26 February 2020	Published	23 January 2020–18 February 2020	China	Wuhan, Hubei	Paediatric inpatients with COVID-19 infection confirmed by pharyngeal swab in Wuhan Children’s Hospital	Cohort	18	65	2*	No	Statistic used for LOS not completely clear. Two neonates remain under observation but had negative CT findings.
Xiao et al. [82]	08 April 2020	Pre-print	05 January 2020–08 March 2020	China	Wuhan, Hubei	Hankou Hospital, COVID-19 confirmed patients ≥18 years, entire stay in hospital ≥48 h, not undergone renal replacement therapy (RRT) before admission	Cohort	287	55.7	62 (51–70)*	No	Not clear whether LOS estimate includes still-hospitalised patients, or only those who are discharged/dead.
Xie et al. [83]	02 April 2020	Published	02 February 2020–23 February 2020	China	Wuhan, Hubei	All laboratory-confirmed COVID-19 discharged patients from the non-ICU ward at Wuhan Jinyintan Hospital	Cohort	79	55.7	60 (48–66)*	Yes	Non-ICU LOS reported. Study dates are based on discharge dates, not admission dates.
Xu et al. [84]	26 March 2020	Pre-print	01 January 2020–20 February 2020	China	Hubei/Anhui	Patients with confirmed COVID-19 admitted to Union Hospital of Huazhong University of Science and Technology, and COVID-19 patients admitted to the Second People’s Hospital of Fuyang City, in Anhui province.	Cohort	355	54.4		No	Not clear whether patients remain in hospital at the study end. LOS only mentioned amongst those with AKI who died. Sex/age distribution not specified for this subgroup.
Yan et al. [85]	30 March 2020	Pre-print	31 January 2020–09 March 2020	China	Hubei	Hospitalised, non-critically ill patients with COVID-19 admitted to No. 3 People’s Hospital of Hubei Province that had the available RNA viral data to estimate the duration of viral shedding.	Cohort	120	45	52 (35–63)*	No	Investigating viral shedding so only included patients with RNA viral data. Most patients (99.2%) in the cohort were non-critically ill patients with COVID-19 due to triage strategies. Had 168 patients admitted over the study period, only reported LOS for 120 that had been discharged.
Yang et al. [54]	24 February 2020	Published	24 December 2019–09 February 2020	China	Wuhan, Hubei	Critically ill adult patients with SARS-CoV-2 pneumonia who were admitted to the intensive care unit (ICU) of Wuhan Jin Yin-Tan Hospital	Cohort	52	67	59 ·7 ± 13 ·3**	Yes	LOS only presented for those that have died.
Yin et al. [40]	11 April 2020	Pre-print	28 January 2020–08 March 2020	China	Wuhan, Hubei	Female inpatients (20–40 years old, female) from January 28 to February 28, 2020, at Wuhan Union and Tongji hospitals of Huazhong University of Science and Technology	Cohort	66	0	20–40	No	Only include pregnant women. Not clear whether patients still-hospitalised were included in the LOS estimate, we have assumed that still-hospitalised patients were included.
Yuan et al. [86]	29 March 2020	Published	11 January 2020–13 February 2020	China	Shenzhen, Guangdong	Confirmed COVID-19 patients which were admitted and subsequently discharged from Shenzhen Third People’s Hospital.	Cohort	94	44.7	40 (1–78)*	Yes	Study dates in abstract and main text differ (abstract: 5 January to 13 February; main text: 11 January to 4 February with follow-up to 13 February)
Zeng et al. [87]	11 April 2020	Pre-print	05 January 2020–08 March 2020	China	Wuhan, Hubei	Hankou Hospital, patients ≥18 years, entire stay in hospital ≥48 h. Confirmed cases of COVID-19	Cohort	274	55	60 ± 15**	No	Excluding short hospital stays (<less 48 h). Not clear whether LOS estimate includes still-hospitalised patients, or only those who are discharges/dead.
Zhang et al. [88]	06 March 2020	Pre-print	02 January 2020–15 February 2020	China	Wuhan, Hubei	Confirmed COVID-19 patients admitted to Zhongnan Hospital of Wuhan University	Cohort	32	48.9	55 (39–66.5)*	No	Patients still hospitalised at the end of the study.
Zhang et al. [41]	26 March 2020	Pre-print		China	Shaanxi	Confirmed COVID-19 patients admitted to Xi’an No. 8 Hospital (Shaanxi Provincial Infectious Disease Hospital) and the First Affiliated Hospital of Xi’an Jiaotong University	Cohort	25			No	Does not describe patient characteristics and statistic for non-ICU LOS not specified
Zhao et al. [89]	30 March 2020	Pre-print	21 January 2020–29 February 2020	China	Beijing	Laboratory-confirmed COVID-19 in Beijing YouAn Hospital, Beijing	Cohort	77	44.2	52 ± 20**	No	Patients still hospitalised at the end of the study.
Zhou et al. [58]	11 March 2020	Published	29 December 2019–31 January 2020	China	Wuhan, Hubei	All adult (≥18 years) inpatients who were hospitalised for COVID-19 (diagnosed with COVID-19 according to WHO interim guidance) and had a definite outcome (dead or discharged), at Jinyintan Hospital and Wuhan Pulmonary Hospital	Cohort	191	62	56 (46–67)*	Yes	The study dates are based on discharge dates and not admission dates. Before 11 January 2020, SARS-CoV-2 RNA detection results were not available in the electronic medical records, from which data for this study were obtained retrospectively; therefore, this study includes 29 of the 41 patients originally reported on.

*Median (IQR); **mean ± SD; ***range

The studies were carried out between 24 December 2019 and 16 April 2020. Although the cut-off was 12 April 2020 for inclusion of published and pre-print studies, the most recent version of the ICNARC report [28] was used, which included patients admitted up to 16 April 2020. The majority of studies were cohort studies (46/52), with four cross-sectional studies, one case-control, and one randomised control trial (RCT). Two articles were reports from ongoing data collections (ISARIC [27], 8 April 2020, and ICNARC [28], 16 April 2020).

Studies were mostly conducted in adults with average participant age from 19 to 76 years (mean (SD) across studies, weighted by sample size, 59 (9.6) years), and overall reported only a slightly higher proportion of males to females (54 (10.9) % male). Three paediatric studies included patients from newborn to 18 years, with a weighted mean (SD) of 7 (2.8) years of age [29–31]. For the majority of studies, LoS was a secondary or incidental outcome rather than the primary outcome. As a result, age and sex distributions were not always specific to the LoS population, and instead reported for the overall study population. Furthermore, it was not always possible to accurately interpret the sample size of the population, nor whether the LoS estimate included still-hospitalised patients. All LoS data extracted from studies are reported in Supplementary Table B.

The majority of the included studies (46/52) were based in China, with a particularly high number reported from Wuhan (27/46), and many study populations were from the same outside of China: there was one study from Italy [32], one for the whole EU region [33], two from the USA [34, 35], one from the UK [28], and one study that collated LoS estimates from multiple countries excluding China (although the majority of the data are from the UK; [27]).

Most studies (43/52) reported LoS for total hospitalisation only, with four studies reporting LoS for ICU only, and five studies reporting both. Only 15 studies reported LoS for study populations with completed follow-up (patient discharge or death), with 37 reporting estimates for populations where some patients remained in hospital or in ICU. The majority of studies only included discharged or dead patients within their LoS estimate, even if they had incomplete follow-up of the full cohort. However, for 8 studies, it was unclear whether the reported LoS included patients who were still hospitalised [33, 34, 36–41].

### Total hospital length of stay

**Fig. 2:**
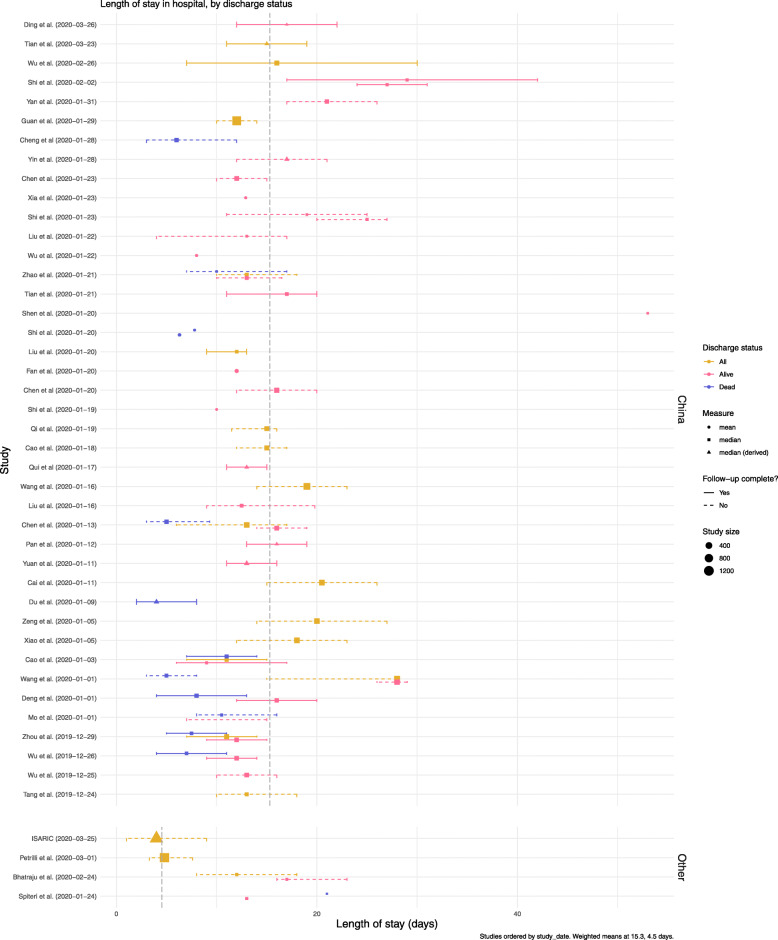
Hospital length of stay, by discharge status. Medians (square) are presented with interquartile range (IQR). Where estimates were reported as mean and standard deviation, equivalent quantiles have been calculated assuming a Weibull distribution (triangle); if no measure of variation was reported, only the original mean is presented (circle). The grey dashed lines represent the mean value across all point estimates within that setting, weighted by sample size. The studies are ordered by the study start date, with most recent at the top. Two studies (Shi et al. (2020-02-02) and Shi et al. (2020-01-23)) have multiple estimates for the same outcome which represent multiple treatment and comorbidity subgroups, respectively. Details of these are included in Table 1

Estimates of the total hospital LoS are summarised in Fig. 2. Where provided, the overall study estimate of LoS for each discharge status is presented. For three studies, LoS was only reported within specific patient subgroups (relating to cardiac injury [42], COVID-19 recovery trajectory [43], and treatment comparison arms [44]); therefore, in these cases, we include both estimates. The longest stays were recorded in a study of five critically ill patients [45], of whom only three were discharged and all more than 50 days after admission, which does not appear representative of the overall distribution (see Fig. 2, Shen et al. (2020-01-20)). Excluding this study, the median duration of hospitalisation ranged from 5 to 29 days. There was no observed trend with respect to when the study was conducted (Fig. 2).

Estimates for LoS amongst patients who died in hospital were generally shorter than those for patients who were discharged alive, with medians between 4 and 21 days compared to 4 and 53 days, respectively. This difference is apparent in Fig. 2, where median LoS was lower for those discharged alive in 6 out of 8 studies that reported both outcomes. In studies that reported total hospital LoS by disease severity (11 studies, Supplementary Fig. A), there was a trend towards more severe cases having longer LoS. However, the definition of different levels of severity was inconsistent between studies so it is not possible to draw any confident conclusion.

Visual inspection of the study estimates suggested some evidence of a difference between total hospital LoS reported within and outside China, but studies outside China were too few (5/48) for a formal comparison. However, LoS reported within the ISARIC report [27] in particular (which includes contributed data from 25 countries, but with the majority of patients from the UK) gave a median and IQR (4 days (1–9)) substantially lower than the weighted mean from the studies from China (15.3 days).

The patient populations observed in these studies covered a wide range of ages, including three paediatric studies [29–31]. Amongst patients discharged alive, there appears to be little difference in average LoS between studies with the youngest and oldest patients, but the longest estimates came from studies with average age in the upper end of the range (Wang et al. [39] and Shi et al. [44], with average age of 68 and 69, respectively; Supplementary Fig. B). The LoS estimates which included non-survivors tended to come from studies with older populations, as is to be expected given the well-documented, age-dependent fatality rate [46].

### ICU length of stay

**Fig. 3:**
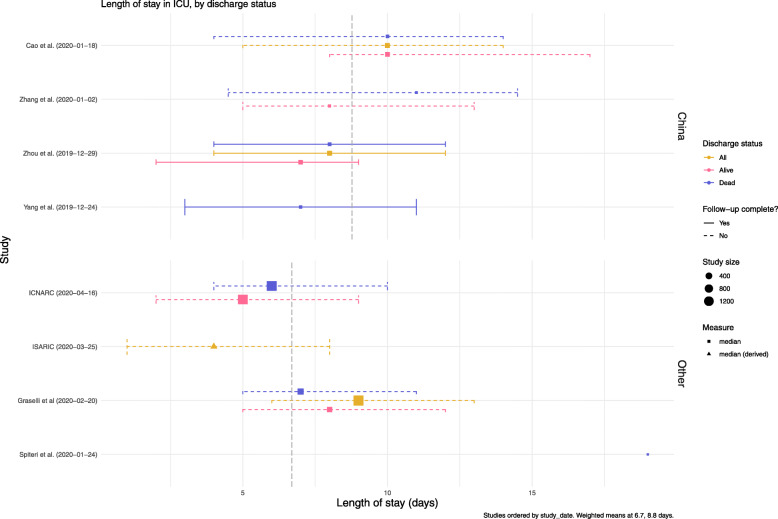
ICU length of stay, by discharge status. Medians (square) are presented with interquartile range (IQR). Where estimates were reported as mean and standard deviation, equivalent quantiles have been calculated assuming a Weibull distribution (triangle); if no measure of variation was reported, only the original mean is presented (circle). The grey dashed lines represent the mean value across all point estimates within that setting, weighted by sample size. Studies are ordered by the study start date

Median stay in ICU ranged from 5 (IQR 2–9) to 19 (no IQR reported) days. There appeared to be less of a difference according to discharge status (alive or dead) than there was for total LoS (Fig. 3). A total of 8 studies reported ICU LoS estimates, with the same number of studies reporting LoS estimates from China and outside of China, and the resulting overall estimates are very similar. There were too few studies to conduct any comparison by age or disease severity.

### Estimated distributions

**Fig. 4:**
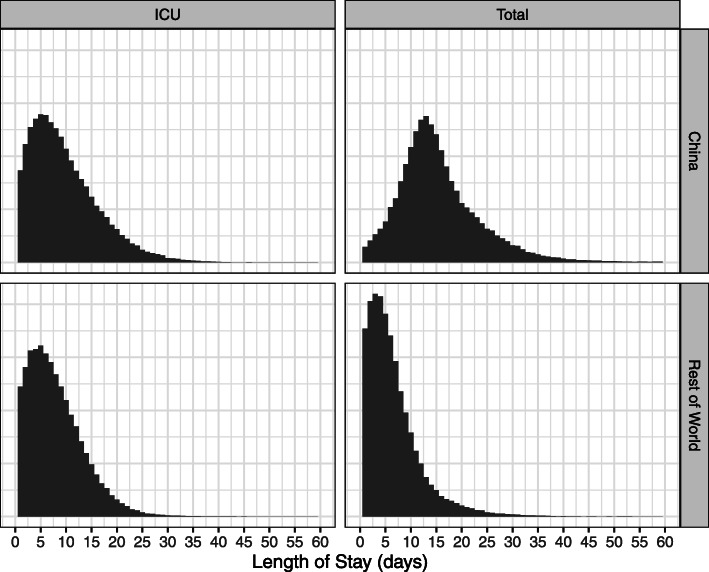
Combined LOS distributions. Samples from the LoS distributions, split by location (China or rest of world) and type (ICU vs total LoS). For each subset, 100,000 draws were taken. The *x*-axis was cut at days = 60

Estimated summary hospital LoS distributions for studies from China and studies outside China are shown in Fig. 4. The median and IQR for total hospital was estimated to be 14 (10–19) for China and 5 (3–9) excluding China. This was also repeated for ICU LoS, with a median and IQR 8 (5–13) for China and 7 (4–11) outside China. Comparing patient outcomes for total LoS, patients who died had a shorter LoS distribution (median, 8; IQR, 4–12) compared with patients who were discharged (median, 14; IQR, 11–17; Supplementary Fig. C). Studies from China which had complete follow-up with respect to total hospital LoS were compared with studies with incomplete follow-up. A slight difference was observed, with shorter median LoS observed in studies with complete follow-up (median, 12; IQR, 8–17) compared with incomplete follow-up (median, 14; IQR, 10–19; Supplementary Fig. D). This was only performed for total hospital LoS in China, since no studies from outside China reported completed LoS for ICU. In addition, LoS estimates of all studies were compared with estimates where overlapping patient populations were removed (Supplementary Fig. E). A list of studies included in this analysis is presented in Supplementary table B. A small difference was observed, with longer LoS estimates observed in studies where overlapping populations had been removed (*n*=17; median, 16; IQR, 11–22) compared with estimates across all studies (*n*=19; median, 14; IQR, 10–19). Again, this sensitivity analysis was only performed for total hospital LoS in China, as it was the largest group.

For total hospital LoS in China, five studies were not included since they only provided point estimates for the LoS. The point estimates from four of these studies fell within the IQR of the estimated distribution; however, for [45], the point estimate was much longer. For total LoS outside of China, one study reported only a point estimate [33], and this also fell outside of the estimated IQR. Sensitivity analysis showed that weighting by sample size had minimal influence on the shape of these distributions (Supplementary Fig. F).

## Discussion

### Summary of findings

Understanding how long patients hospitalised with COVID-19 remain in hospital is critical for planning and predicting bed occupancy as well as associated staff and equipment needs. This review found that hospital LoS observations for COVID-19 patients published in the literature to date varied from less than a week to nearly 2 months. Stay in intensive care was shorter and less variable, with studies reporting medians of 1 to 3 weeks. Where LoS was reported according to discharge status, stay was found to be shorter for those who died than for those discharged alive; however, this difference was only apparent in terms of total stay and not stay in ICU (no statistical comparison was made). With respect to practical implications, knowledge of a difference between survivors and non-survivors is of less use since the outcome will not be known in advance in order to influence decision-making. To the authors’ knowledge, this is the first formal review that has been conducted on hospital LoS for COVID-19.

The included studies yield some evidence of a difference between total hospital LoS observed in China and outside of China, with shorter LoS reported in the latter group (14 days (10–19) vs 5 days (3–9), respectively). However, only five studies were identified which reported LoS outside of China; therefore, this comparison is somewhat inconclusive. It may be that LoS is longer in China compared with other settings due to different criteria for hospital admission and discharge. A consensus exists across guidelines, such as ensuring resolution of symptoms and evidence of two negative PCR samples at least 24 h apart before discharge [47, 48]; however, differences between settings may arise as a result of local capacity and strain on the health system. We attempted to capture this difference by recording time from onset of symptoms to admission; however, only one study outside of China reported this and a comparison was not possible. It is also possible that, with foresight from witnessing the Chinese epidemic, other countries set less strict criteria for discharge, in anticipation of stretched capacity. Other countries may also have used evidence from China to improve treatment methods and hence shorten LoS. However, this unfortunately appears unlikely as we did not observe a trend when looking at the reported LoS estimates over time.

In contrast, no difference was observed between settings for ICU LoS, for which there were an equal number of studies included from within and outside China. It is important to note that there might be key differences between ICUs in China compared with other countries, yet a definition for what constituted an ICU was rarely reported. Previous studies have found that ICU characteristics varied widely across geographic regions [49]. Further understanding of characteristics of ICUs reporting LoS for patients with COVID-19 is important in providing context on the reported estimates and should be investigated in future studies.

There appeared to be little difference of LoS observed by age in our results, apart from the fact that studies which reported deaths tended to have older patient populations. However, if there is indeed a trend, we were unlikely to observe strong evidence for it amongst these studies, since the majority include a similar mix of ages, often tending towards older cohorts, and the age distribution was not always provided for the specific subgroup who had LoS recorded. Two studies [27, 38] were included in the review which reported LoS by age, and they both found longer LoS associated with older age groups. In addition, two studies of LoS from the USA which were published after the search dates also reported a trend for longer LoS in older age groups [50, 51]. Studies also reported LoS by disease severity; however, the definitions of disease severity were not consistent across studies, so we did not summarise by this.

### Limitations and biases

Having been the first country to observe this novel coronavirus, published data on COVID-19 patient outcomes in China is more widely available than from countries to which the epidemic spread later on. The set of studies found in this review reflects this bias towards evidence obtained from China, particularly Wuhan. The small number of studies identified from outside of China means it is difficult to interpret comparisons across settings. Several studies have been published after our search dates which provide additional LoS estimates from outside of China. A study of 5700 patients from hospitals in the New York area reported comparable estimates for total LoS (median 4.5; IQR 2.4–8.1) [51]; however, studies from Northern Italy [52], Japan [53], and California and Washington [50] reported longer estimates of LoS. Therefore, the total LoS outside of China may in fact be longer than what we concluded. Our code is freely available on github, and additional studies may easily be added. As more studies emerge from a broader range of settings, it would be important to re-evaluate LoS estimates, as there are likely to be between-country differences that we have not captured here.

Furthermore, a number of studies include patients from the same hospital over the same period, for example, Yang et al. [54] and Wu et al. [55] who both reported patients from Jin Yin-Tan hospital in Wuhan, and it is possible that these studies had overlapping study populations. Furthermore, Guan et al. [36] was a national study conducted in China and ISARIC [27] included 25 countries worldwide; therefore, these studies may also include patients previously described. The effect of this double-counting would be to bias the overall summary statistics towards the LoS from these settings, and potentially reduce the total variation. Although this is acknowledged as an issue, it was not considered as a basis for exclusion since any criteria for selecting one study from the overlapping group would have been arbitrary and potentially induce another source of bias in itself. Therefore, we instead chose to conduct a sensitivity analysis based on a straightforward selection criteria of largest sample size from those studies with any potential for overlap, and found little difference between the estimates of LoS whether overlapping studies were included or excluded. The overall benefit of inclusion, particularly as many of these studies reported LoS for different subgroups, was deemed to outweigh the potential bias which may arise as a result of overlapping patient populations.

In this review, we were only able to distinguish between “total hospital LoS” and “ICU LoS”, with many studies only reporting a total LoS. This total LoS will include both general hospital and ICU admissions within it. There is a need for more granularity with respect to patient pathways, distinguishing between admissions to different levels of care within one hospital episode in order to better inform healthcare contingencies. Patients may, for example, be transferred to ICU on more than one occasion during their stay, which is important to factor in when ICU capacity is particularly limited.

Changes in hospital demand may have also affected our estimates. At the beginning of the outbreak and in certain settings, hospitals were being used to isolate patients who were unable to isolate effectively at home [11, 12]. This means that LoS for patients in some of the earlier studies within this dataset could have been longer due to this logistical reason, rather than clinical need. Studies which mentioned this explicitly were excluded, yet there may still be others which were not so transparent. In addition, it is possible that, as hospitals reach the limits of their capacity, a more stringent triage policy may be implemented and the most critical patients may not be transferred to ICU. Despite this, we did not observe a trend when looking at the reported LoS estimates over time, suggesting that this is not in fact an important issue in our data.

Finally, many studies had incomplete follow-up with respect to LoS, and as a result, patients still hospitalised at the end of the study were not included in the summary statistics (right-truncation). This will bias estimates towards shorter LoS, as patients with longer LoS will not be included. A study by Lapidus et al. [56] investigated the bias associated with estimating average ICU LoS for COVID-19 patients based on observed LoS of discharged patients before follow-up of the entire patient cohort was completed. As expected, the authors found that the average LoS estimated at 3 months of follow-up was much longer than that estimated at 1 month. This potentially affects our estimates, given that 37 (out of 52) studies had incomplete follow-up with regard to LoS, although on comparison the difference between the groups was slight, and estimates where follow-up was complete were overall shorter. Several studies included still-hospitalised patients in their LoS summary without accounting for censoring [33, 34, 36–41], which potentially alters interpretation of the values.

### Summarising length of stay

We found that LoS is often not the primary measure of interest in studies which report it; however, it is an important parameter when it comes to forecasting bed occupancy during an outbreak. By conducting this review, we have systematically gathered a range of published estimates, providing a source from which researchers and decision-makers can obtain estimates specific to their population of interest (e.g. with respect to comorbidities) and allowing comparison of LoS between several different populations and settings.

There have been numerous previous studies which have aimed to forecast the number of hospital beds required for COVID-19 patients [16–22, 57]. Many of these studies published so far have used point estimates, only originating from one study which often does not reflect the context of interest. In particular, many used estimates from Zhou et al. [58] which reported a shorter total hospital LoS (median 11 ·0, IQR 7 ·0–14 ·0; Fig. 2), and a comparable ICU LoS (median 8 ·0, IQR 4 ·0–12 ·0; Fig. 3), compared with other studies from China. However, both of these were still longer than LoS estimates reported by studies outside of China. This means that the bed-forecasting studies relying on LoS estimates from Zhou et al. may be underestimating the number of beds required. This review has highlighted several potential sources of variation in LoS and identified common issues and biases which influence each individual estimate. This gives a motivation for considering a wider range of values than can be obtained in a single study, aiming instead to capture the overall distribution of LoS across a variety of possible patient trajectories.

Our findings provide both better estimates of the LoS distribution for patients hospitalised with COVID-19 and a method for generating these important distributions going forward. Combined with predictions of disease incidence, models forecasting bed occupancy are used to plan required hospital capacity and hence are critical for outbreak preparations. The LoS estimate is a critical parameter within such a model, and as such, any predictions are sensitive to the value or distribution being assumed, with strong implications for policy and planning. In particular, the tail of the LoS distribution must not be ignored since these few patients can block beds for a long time and form a heavy burden on capacity. Our estimates, and the proposed method for distribution collation, allow for improved predictions of this aspect of burden prediction and hence could reduce uncertainty in capacity preparedness in healthcare settings going forward. This is of value to countries still experiencing growing epidemics and those turning attention to the planning for the possibility of a second wave whilst restarting non-COVID care.

It is preferable to use data from the setting for which you are trying to forecast bed occupancy (as was done by the IHME COVID-19 health service utilisation forecasting team [15]); however, data on completed patient stays will often not be available until well after the onset of the epidemic. Furthermore, LMICs may have reduced capacity for surveillance and monitoring in order to obtain these data. In such cases, where countries are in the early stages of an outbreak, it would be better to use a conservative (i.e. broad) distribution of LoS from another setting. As the pandemic progresses and more countries observe patients completing their hospital episodes, it will be possible to add further setting-specific summaries and improve this distribution.

As far as the authors are aware, the approach demonstrated here to summarise median and IQRs across multiple studies has not been proposed before, although there are similarities with the approach taken by others in the CMMID Working Group to pool *R*_0_ estimates [59]. We present an intuitive method which exploits two optimisation methods to fit parametric distributions based on reported summary statistics rather than individual data, then samples across them. In this way, we capture the central tendency and overall variation between a set of quantiles from different study populations. This allows multiple sources of evidence to be consolidated into a single distribution which can be used in bed forecasting going forward. By providing both the code for this analysis and our summary distributions, better bed occupancy predictions can be made in the future.

## Conclusion

This review summarised the available literature to provide estimates of LoS for total hospital admission and ICU which can be applied for planning and preparedness for SARS-CoV-2. We found substantial differences between China and other settings in terms of total hospital stay, but little evidence for an impact on LoS of time of study, age, or disease severity. We present summary distributions which can be used within models making predictions about bed requirements, and suggest that this may be a more robust and realistic way to characterise LoS than relying on summary data from just one setting or hospital. The majority of the data presented in this review comes from China, and as more data become available, it will be important to update this with setting-specific LoS estimates. Understanding the duration of hospitalisation of COVID-19 patients is critical for providing insights as to when hospitals will reach capacity, as well predicting associated staff or equipment requirements.

## Supplementary information

**Additional file 1** List of search terms. Supplementary Table A. Description of information extracted from included studies. Fig SA: LoS by disease severity. Fig SB: LoS by study median age. Fig SC: LoS by outcome status. Fig SD: Summary distributions for China/other and total/ICU. Fig SE: Sensitivity of length of stay (LoS) with and without overlapping populations removed. Fig SF: Sensitivity analysis of summary distributions to weighting.

**Additional file 2** Supplementary Table B. Database of all studies included within the review. Available as an excel spreadsheet.

## Data Availability

The data and code used in this work can be accessed at https://github.com/esnightingale/los_review.

## Abbreviations

LoS: Length of stay
ICU: Intensive care unit
IQR: Interquartile range
SD: standard deviation
LMIC: low- and middle-income country
PRISMA: Preferred Reporting Items for Systematic Reviews
RCT: randomised control trial.

## References

[1] World Health Organization. Coronavirus disease 2019 (COVID-19) situation report, 95. 2020. Available from: https://www.who.int/docs/defaultsource/coronaviruse/situation-reports/20200424-sitrep-95-covid-19.pdf?sfvrsn=e8065831\_4.

[2] Xie J, Tong Z, Guan X, Du B, Qiu H, Slutsky AS. Critical care crisis and some recommendations during the COVID-19 epidemic in China. Intensive Care Med. 2020; 46:837–40. Available from: 10.1007/s00134-020-05979-7. 10.1007/s00134-020-05979-7 PMC7080165 32123994

[3] Qiu H, Tong Z, Ma P, Hu M, Peng Z, Wu W, Du B. Intensive care during the coronavirus epidemic. Intensive Care Med. 2020; 46(4):576–8. Available from: 10.1007/s00134-020-05966-y. 10.1007/s00134-020-05966-y PMC7080064 32077996

[4] Remuzzi A, Remuzzi G. COVID-19 and Italy: what next?Lancet. 2020; 395(10231):1225–8. Available from: https://www.thelancet.com/journals/lancet/article/PIIS0140-6736(20)3062%7-9/abstract. 10.1016/S0140-6736(20)30627-9 PMC7102589 32178769

[5] Paterlini M. On the front lines of coronavirus: the Italian response to covid-19. BMJ. 2020; 368:m1065. Available from: https://www.bmj.com/content/368/bmj.m1065. 10.1136/bmj.m1065 32179517

[6] Legido-Quigley H, Mateos-Garciá JT, Campos VR, Gea-Sánchez M, Muntaner C, McKee M. The resilience of the Spanish health system against the COVID-19 pandemic. Lancet Public Health. 2020; 5(5):251–2. Available from: https://www.thelancet.com/journals/lanpub/article/PIIS2468-2667(20)3006%0-8/abstract. 10.1016/S2468-2667(20)30060-8 PMC7104264 32199083

[7] Rosenbaum L. Facing Covid-19 in Italy?ethics, logistics, and therapeutics on the epidemic’s front line. N Engl J Med. 2020; 382(20):1873–5. Available from: 10.1056/NEJMp2005492. 10.1056/NEJMp2005492 32187459

[8] Rodriguez-Morales AJ, Cardona-Ospina JA, Gutiérrez-Ocampo E, Villamizar-Peña R, Holguin-Rivera Y, Escalera-Antezana JP, Alvarado-Arnez LE, Bonilla-Aldana DK, Franco-Paredes C, Henao-Martinez AF, et al. Clinical, laboratory and imaging features of COVID-19: a systematic review and meta-analysis. Travel Med Infect Dis. 2020:101623. Available from: http://www.sciencedirect.com/science/article/pii/S1477893920300910. 10.1016/j.tmaid.2020.101623 PMC7102608 32179124

[9] Cascella M, Rajnik M, Cuomo A, Dulebohn SC, Di Napoli R. Features, evaluation and treatment coronavirus (COVID-19). In: StatPearls. Treasure Island (FL). StatPearls Publishing: 2020. Available from: http://www.ncbi.nlm.nih.gov/books/NBK554776/. 32150360

[10] Jiang F, Deng L, Zhang L, Cai Y, Cheung CW, Xia Z. Review of the clinical characteristics of coronavirus disease 2019 (COVID-19). J Gen Intern Med. 2020:1–5. Available from: 10.1007/s11606-020-05762-w. 10.1007/s11606-020-05762-w PMC7088708 32133578

[11] Chen S, Zhang Z, Yang J, Wang J, Zhai X, Bärnighausen T, Wang C. Fangcang shelter hospitals: a novel concept for responding to public health emergencies. Lancet. 2020; 395(10232):1305–14. Available from: https://www.thelancet.com/journals/lancet/article/PIIS0140-6736(20)30744-3. 10.1016/S0140-6736(20)30744-3 PMC7270591 32247320

[12] Wu Z, McGoogan JM. Characteristics of and important lessons from the coronavirus disease 2019 (COVID-19) outbreak in China: summary of a report of 72 314 cases from the Chinese Center for Disease Control and Prevention. JAMA. 2020; 323(13):1239–42. Available from: https://jamanetwork.com/journals/jama/fullarticle/2762130. 10.1001/jama.2020.2648 32091533

[13] Yang J, Zheng Y, Gou X, Pu K, Chen Z, Guo Q, Ji R, Wang H, Wang Y, Zhou Y. Prevalence of comorbidities and its effects in coronavirus disease 2019 patients: a systematic review and meta-analysis. Int J Infect Dis. 2020; 94:91–5. Available from: http://www.sciencedirect.com/science/article/pii/S1201971220301363. 10.1016/j.ijid.2020.03.017 PMC7194638 32173574

[14] Clark A, Jit M, Warren-Gash C, Guthrie B, Wang HH, Mercer SW, Sanderson C, McKee M, Troeger C, Ong KI, et al. How many are at increased risk of severe COVID-19 disease? Rapid global, regional and national estimates for 2020. medRxiv. 2020. Available from: https://www.medrxiv.org/content/10.1101/2020.04.18.20064774. 10.1016/S2214-109X(20)30264-3 PMC7295519 32553130

[15] COVID IHME, Murray CJ. Forecasting COVID-19 impact on hospital bed-days, ICU-days, ventilator-days and deaths by US state in the next 4 months. Infect Dis (except HIV/AIDS). 2020. Available from: http://medrxiv.org/lookup/doi/10.1101/2020.03.27.20043752.

[16] Deasy J, Rocheteau E, Kohler K, Stubbs DJ, Barbiero P, Liò P, Ercole A. Forecasting ultra-early intensive care strain from COVID-19 in England. medRxiv. 2020. Available from: http://medrxiv.org/content/early/2020/04/07/2020.03.19.20039057.

[17] Ferstad JO, Gu AJ, Lee RY, Thapa I, Shin AY, Salomon JA, Glynn P, Shah NH, Milstein A, Schulman K, Scheinker D. A model to forecast regional demand for COVID-19 related hospital beds. medRxiv. 2020. Available from: http://medrxiv.org/content/early/2020/04/07/2020.03.26.20044842.

[18] Weissman GE, Crane-Droesch A, Chivers C, Luong T, Hanish A, Levy MZ, Lubken J, Becker M, Draugelis ME, Anesi GL, et al. Locally informed simulation to predict hospital capacity needs during the COVID-19 pandemic. Ann Intern Med. 2020; 173(1):21–8. Available from: 10.7326/M20-1260. 10.7326/M20-1260 PMC7153364 32259197

[19] Massonnaud C, Roux J, Crépey P. COVID-19: forecasting short term hospital needs in France. medRxiv. 2020. Available from: http://medrxiv.org/content/early/2020/03/20/2020.03.16.20036939.

[20] Moghadas SM, Shoukat A, Fitzpatrick MC, Wells CR, Sah P, Pandey A, Sachs JD, Wang Z, Meyers LA, Singer BH, et al. Projecting hospital utilization during the COVID-19 outbreaks in the United States. Proc Natl Acad Sci. 2020; 117(16):9122–6. Available from: https://www.pnas.org/content/117/16/9122. 10.1073/pnas.2004064117 PMC7183199 32245814

[21] Shoukat A, Wells CR, Langley JM, Singer BH, Galvani AP, Moghadas SM. Projecting demand for critical care beds during COVID-19 outbreaks in Canada. CMAJ. 2020; 192(19):489–96. Available from: https://www.cmaj.ca/content/early/2020/04/09/cmaj.200457. 10.1503/cmaj.200457 PMC7234264 32269020

[22] Castro MC, Carvalho LR, Chin T, Kahn R, Franca GVA, Macario EM, de Oliveira WK. Demand for hospitalization services for COVID-19 patients in Brazil. medRxiv. 2020. Available from: https://www.medrxiv.org/content/10.1101/2020.03.30.20047662.

[23] Marazzi A, Paccaud F, Ruffieux C, Beguin C (1998). Fitting the distributions of length of stay by parametric models. Med Care.

[24] R Core Team. R stats package version 1.14.4.Available from: https://www.rdocumentation.org/packages/stats/versions/3.6.2.

[25] Macdonald P, Du J. Mixdist: finite mixture distribution models version 0.5-5. 2018. Available from: https://CRAN.R-project.org/package=mixdist.

[26] Locke S, FitzJohn R, Cori A, Jombart T. distcrete: Discrete Distribution Approximations version 1.0.3. 2017. Available from: https://CRAN.Rproject.org/package=distcrete.

[27] International Severe Acute Respiratory and Emerging Infections Consortium (ISARIC). COVID-19 report. 2020. Available from: https://media.tghn.org/medialibrary/2020/04/ISARIC_Data_Platform_COVID-%19_Report_8APR20.pdf.

[28] Intensive Care National Audit and research Centre (ICNArC). Report on 2249 patients critically ill with COVID-19. 2020. Available from: https://www.icnarc.org/Our-AuditLatest-News2020/04/04/report-On-2249-Patients-Critically-Ill-With-Covid-19.

[29] Qiu H, Wu J, Hong L, Luo Y, Song Q, Chen D. Clinical and epidemiological features of 36 children with coronavirus disease 2019 (COVID-19) in Zhejiang, China: an observational cohort study. Lancet Infect Dis. 2020; 20(6):689–96. Available from: https://www.thelancet.com/journals/laninf/article/PIIS1473-3099(20)3019%8-5. 10.1016/S1473-3099(20)30198-5 PMC7158906 32220650

[30] Shi Y, Wang X, Liu G, Zhu Q, Wang J, Yu H, Wang C, Wang L, Zhang M, Zhang L, et al. A quickly, effectively screening process of novel corona virus disease 2019 (COVID-19) in children in Shanghai, China. Ann Transl Med. 2020; 8(5):241. Available from: http://atm.amegroups.com/article/view/37468. 10.21037/atm.2020.03.22 PMC7154461 32309388

[31] Xia W, Shao J, Guo Y, Peng X, Li Z, Hu D. Clinical and CT features in pediatric patients with COVID-19 infection: different points from adults. Pediatr Pulmonol. 2020; 55(5):1169–74. Available from: https://onlinelibrary.wiley.com/doi/abs/10.1002/ppul.24718. 10.1002/ppul.24718 PMC7168071 32134205

[32] Grasselli G, Zangrillo A, Zanella A, Antonelli M, Cabrini L, Castelli A, Cereda D, Coluccello A, Foti G, Fumagalli R, et al. Baseline characteristics and outcomes of 1591 patients infected with SARS-CoV-2 admitted to ICUs of the Lombardy Region, Italy. JAMA. 2020; 323(16):1574–81. Available from: 10.1001/jama.2020.5394. 10.1001/jama.2020.5394 PMC7136855 32250385

[33] Spiteri G, Fielding J, Diercke M, Campese C, Enouf V, Gaymard A, Bella A, Sognamiglio P, Moros MJS, Riutort AN, et al. First cases of coronavirus disease 2019 (COVID-19) in the WHO European Region, 24 January to 21 February 2020. Eurosurveillance. 2020; 25(9):2000178. Available from: https://www.eurosurveillance.org/content/10.2807/1560-7917.ES.2020.25.9.2000178. 10.2807/1560-7917.ES.2020.25.9.2000178 PMC7068164 32156327

[34] Bhatraju PK, Ghassemieh BJ, Nichols M, Kim R, Jerome KR, Nalla AK, Greninger AL, Pipavath S, Wurfel MM, Evans L, et al. Covid-19 in critically ill patients in the Seattle region?case series. N Engl J Med. 2020; 382(21):2012–22. Available from: https://www.nejm.org/doi/full/10.1056/NEJMoa2004500. 10.1056/NEJMoa2004500 PMC7143164 32227758

[35] Petrilli CM, Jones SA, Yang J, Rajagopalan H, O’Donnell LF, Chernyak Y, Tobin K, Cerfolio RJ, Francois F, Horwitz LI. Factors associated with hospitalization and critical illness among 4,103 patients with COVID-19 disease in New York City. medRxiv. 2020. Available from: http://medrxiv.org/content/early/2020/04/11/2020.04.08.20057794. 10.1136/bmj.m1966 PMC7243801 32444366

[36] Guan Wj, Ni Zy, Hu Y, Liang Wh, Ou Cq, He Jx, et al.Clinical characteristics of coronavirus disease 2019 in China. N Engl J Med. 2020. Massachusetts Medical Society. Available from: 10.1056/NEJMoa2002032. 10.1056/NEJMoa2002032 PMC7092819 32109013

[37] Liu F, Xu A, Zhang Y, Xuan W, Yan T, Pan K, et al.Patients of COVID-19 may benefit from sustained lopinavir-combined regimen and the increase of eosinophil may predict the outcome of COVID-19 progression. Int J Infect Dis. 2020:S1201971220301326. Available from: https://linkinghub.elsevier.com/retrieve/pii/S1201971220301326. 10.1016/j.ijid.2020.03.013 PMC7193136 32173576

[38] Wang Z, Ji JS, Liu Y, Liu R, Zha Y, Chang X, et al.Survival analysis of hospital length of stay of novel coronavirus (COVID-19) pneumonia patients in Sichuan, China. medRxiv. 2020. Available from: http://medrxiv.org/content/early/2020/04/10/2020.04.07.20057299.abstract.

[39] Wang L, He W, Yu X, Hu D, Bao M, Liu H (2020). Coronavirus disease 2019 in elderly patients: characteristics and prognostic factors based on 4-week follow-up. J Infect.

[40] Yin M, Zhang L, Deng G, Han C, Shen M, Sun H, et al.Severe acute respiratory syndrome coronavirus 2 (SARS-CoV-2) infection during pregnancy in China: a retrospective cohort study. medRxiv. 2020. Available from: http://medrxiv.org/content/early/2020/04/11/2020.04.07.20053744.abstract.

[41] Zhang D, Guo R, Lei L, Liu H, Wang Y, Wang Y, et al.COVID-19 infection induces readily detectable morphological and inflammation-related phenotypic changes in peripheral blood monocytes, the severity of which correlate with patient outcome. medRxiv. 2020. Available from: http://medrxiv.org/content/early/2020/03/26/2020.03.24.20042655.abstract.

[42] Shi S, Qin M, Shen B, Cai Y, Liu T, Yang F (2020). Association of cardiac injury with mortality in hospitalized patients with COVID-19 in Wuhan, China. JAMA Cardiol.

[43] Shi H, Han X, Jiang N, Cao Y, Alwalid O, Gu J, et al.Radiological findings from 81 patients with COVID-19 pneumonia in Wuhan, China: a descriptive study. Lancet Infect Dis. 2020; 20(4):425–34. Available from: https://www.unboundmedicine.com/medline/citation/32105637/Radiological_findings_from_81_patients_with_COVID_19_pneumonia_in_Wuhan_China:_a_descriptive_study_. 10.1016/S1473-3099(20)30086-4 PMC7159053 32105637

[44] Shi C, Wang C, Wang H, Yang C, Cai F, Zeng F, et al.The potential of low molecular weight heparin to mitigate cytokine storm in severe COVID-19 patients: a retrospective clinical study. medRxiv. 2020. Available from: http://medrxiv.org/content/early/2020/04/15/2020.03.28.20046144.abstract. 10.1111/cts.12880 PMC7719364 32881340

[45] Shen C, Wang Z, Zhao F, Yang Y, Li J, Yuan J, et al.Treatment of 5 critically ill patients with COVID-19 with convalescent plasma. JAMA. 2020. Available from: 10.1001/jama.2020.4783. 10.1001/jama.2020.4783 PMC7101507 32219428

[46] CDC. Coronavirus disease 2019 (COVID-19). 2020. Available from: https://www.cdc.gov/coronavirus/2019-ncov/need-extra-precautions/people-at-higher-risk.html.

[47] for Disease PreventionandControl EC. Discharge criteria for confirmed COVID-19 cases. 2020. Available from: https://www.ecdc.europa.eu/sites/default/files/documents/COVID-19-Discharge-criteria.pdf.

[48] Commission CNH. COVID-19 diagnostic guidelines (version 3). 2020. Available from: http://www.chinacdc.cn/jkzt/crb/zl/szkb_11803/jszl_11815/202001/W020200123581113562555.pdf.

[49] Sakr Y, Moreira CL, Rhodes A, Ferguson ND, Kleinpell R, Pickkers P, et al.The impact of hospital and ICU organizational factors on outcome in critically ill patients: results from the extended prevalence of infection in intensive care study*. Crit Care Med. 2015; 43(3):519–26. Available from: http://journals.lww.com/00003246-201503000-00002. 10.1097/CCM.0000000000000754 25479111

[50] Lewnard JA, Liu VX, Jackson ML, Schmidt MA, Jewell BL, Flores JP, et al.Incidence, clinical outcomes, and transmission dynamics of hospitalized 2019 coronavirus disease among 9,596,321 individuals residing in California and Washington, United States: a prospective cohort study. Epidemiology. 2020. Available from: http://medrxiv.org/lookup/doi/10.1101/2020.04.12.20062943. 10.1136/bmj.m1923 PMC7243800 32444358

[51] Richardson S, Hirsch JS, Narasimhan M, Crawford JM, McGinn T, Davidson KW, et al.Presenting characteristics, comorbidities, and outcomes among 5700 patients hospitalized with COVID-19 in the New York City area. JAMA. 2020; 323(20):2052–2059. Available from: https://jamanetwork.com/journals/jama/fullarticle/2765184. 10.1001/jama.2020.6775 PMC7177629 32320003

[52] Inciardi RM, Adamo M, Lupi L, Cani DS, Di Pasquale M, Tomasoni D, et al.Characteristics and outcomes of patients hospitalized for COVID-19 and cardiac disease in Northern Italy. Eur Heart J. 2020; 41(19):1821–9. Available from: https://academic.oup.com/eurheartj/article/41/19/1821/5834516. 10.1093/eurheartj/ehaa388 PMC7239204 32383763

[53] Zaninotto M, Mion MM, Cosma C, Rinaldi D, Plebani M. Presepsin in risk stratification of SARS-CoV-2 patients. Clin Chim Acta. 2020; 507:161–163. Available from: http://www.sciencedirect.com/science/article/pii/S0009898120301753. 10.1016/j.cca.2020.04.020 PMC7175898 32333860

[54] Yang X, Yu Y, Xu J, Shu H, Xia J, Liu H, et al.Clinical course and outcomes of critically ill patients with SARS-CoV-2 pneumonia in Wuhan, China: a single-centered, retrospective, observational study. Lancet Respir Med. 2020. Elsevier. Available from: https://www.thelancet.com/journals/lanres/article/PIIS2213-2600(20)30079-5/abstract. 10.1016/S2213-2600(20)30079-5 PMC7102538 32105632

[55] Wu C, Hu X, Song J, Du C, Xu J, Yang D, et al.Heart injury signs are associated with higher and earlier mortality in coronavirus disease 2019 (COVID-19). medRxiv. 2020. Available from: http://medrxiv.org/content/early/2020/02/29/2020.02.26.20028589.abstract.

[56] Lapidus N, Zhou X, Carrat F, Riou B, Zhao Y, Hejblum G. Biased and unbiased estimation of the average lengths of stay in intensive care units in the COVID-19 pandemic. medRxiv. 2020. Available from: http://medrxiv.org/content/early/2020/04/24/2020.04.21.20073916.abstract. 10.1186/s13613-020-00749-6 PMC7561433 33063241

[57] Murray CJ. Forecasting COVID-19 impact on hospital bed-days, ICU-days, ventilator-days and deaths by US state in the next 4 months. medRxiv. 2020. Available from: http://medrxiv.org/content/early/2020/03/30/2020.03.27.20043752.abstract.

[58] Zhou F, Yu T, Du R, Fan G, Liu Y, Liu Z (2020). Clinical course and risk factors for mortality of adult inpatients with COVID-19 in Wuhan, China: a retrospective cohort study. Lancet.

[59] Davies NG, Kucharski AJ, Eggo RM, Gimma A, Group CCW, Edmunds WJ. The effect of non-pharmaceutical interventions on COVID-19 cases, deaths and demand for hospital services in the UK: a modelling study. medRxiv. 2020. Available from: https://www.medrxiv.org/content/10.1101/2020.04.01.20049908v1. 10.1016/S2468-2667(20)30133-X PMC7266572 32502389

[60] Cai Q, Huang D, Ou P, Yu H, Zhu Z, Xia Z, et al.COVID-19 in a designated infectious diseases hospital outside Hubei Province, China. Allergy. 2020. Available from: https://onlinelibrary.wiley.com/doi/abs/10.1111/all.14309. 10.1111/all.14309 32239761

[61] Cao B, Wang Y, Wen D, Liu W, Wang J, Fan G (2020). A trial of lopinavir-ritonavir in adults hospitalized with severe covid-19. N Engl J Med.

[62] Cao J, Tu WJ, Cheng W, Yu L, Liu YK, Hu X, et al.Clinical features and short-term outcomes of 102 patients with corona virus disease 2019 in Wuhan, China. Clin Infect Dis. 2020. Available from: https://academic.oup.com/cid/article/doi/10.1093/cid/ciaa243/5814897. 10.1093/cid/ciaa243 PMC7184479 32239127

[63] Chen T, Wu D, Chen H, Yan W, Yang D, Chen G, et al.Clinical characteristics of 113 deceased patients with coronavirus disease 2019 : retrospective study. BMJ. 2020:368. BMJ Publishing Group Ltd. Available from: https://www.bmj.com/content/368/bmj.m1091. 10.1136/bmj.m1091 PMC7190011 32217556

[64] Chen J, Qi T, Liu L, Ling Y, Qian Z, Li T, et al.Clinical progression of patients with COVID-19 in Shanghai, China. J Infect. 2020; 80(5):e1–e6. Available from: https://linkinghub.elsevier.com/retrieve/pii/S0163445320301195. 10.1016/j.jinf.2020.03.004 PMC7102530 32171869

[65] Chen X, Zheng F, Qing Y, Ding S, Yang D, Lei C, et al.Epidemiological and clinical features of 291 cases with coronavirus disease 2019 in areas adjacent to Hubei, China: a double-center observational study. medRxiv. 2020. Available from: http://medrxiv.org/content/early/2020/03/06/2020.03.03.20030353.abstract.

[66] Cheng Y, Luo R, Wang K, Zhang M, Wang Z, Dong L, et al.Kidney impairment is associated with in-hospital death of COVID-19 patients. medRxiv. 2020. Available from: http://medrxiv.org/content/early/2020/02/20/2020.02.18.20023242.abstract. 10.1016/j.kint.2020.03.005 PMC7110296 32247631

[67] Deng Y, Liu W, Liu K, Fang YY, Shang J, Zhou L, et al.Clinical characteristics of fatal and recovered cases of coronavirus disease 2019 (COVID-19) in Wuhan, China: a retrospective study. Chin Med J (Engl). 2020. Publish Ahead of Print. Available from: https://journals.lww.com/cmj/Fulltext/9000/Clinical_characteristics_of_fatal_and_recovered.99319.aspx. 10.1097/CM9.0000000000000824 PMC7289311 32209890

[68] Ding Q, Lu P, Fan Y, Xia Y, Liu M. The clinical characteristics of pneumonia patients coinfected with 2019 novel coronavirus and influenza virus in Wuhan, China. J Med Virol. 2020. Available from: https://onlinelibrary.wiley.com/doi/abs/10.1002/jmv.25781. 10.1002/jmv.25781 PMC7228290 32196707

[69] Du Y, Tu L, Zhu P, Mu M, Wang R, Yang P, et al.Clinical features of 85 fatal cases of COVID-19 from Wuhan: a retrospective observational study. Am J Respir Crit Care Med. 2020. American Thoracic Society - AJRCCM. Available from: https://www.atsjournals.org/doi/10.1164/rccm.202003-0543OC. 10.1164/rccm.202003-0543OC PMC7258652 32242738

[70] Fan Z, Chen L, Li J, Cheng X, Yang J, Tian C, et al.Clinical features of COVID-19-related liver damage. Clin Gastroenterol Hepatol. 2020:S1542356520304821. Available from: https://linkinghub.elsevier.com/retrieve/pii/S1542356520304821. 10.1016/j.cgh.2020.04.002 PMC7194865 32283325

[71] Liu J, Ouyang L, Guo P, Wu HS, Fu P, Chen YL, et al.Epidemiological, clinical characteristics and outcome of medical staff infected with COVID-19 in Wuhan, China: a retrospective case series analysis. medRxiv. 2020. Available from: http://medrxiv.org/content/early/2020/03/13/2020.03.09.20033118.abstract.

[72] Liu L, Gao JY. Clinical characteristics of 51 patients discharged from hospital with COVID-19 in Chongqing???China. medRxiv. 2020. Available from: http://medrxiv.org/content/early/2020/02/23/2020.02.20.20025536.abstract.

[73] Mo P, Xing Y, Xiao Y, Deng L, Zhao Q, Wang H, et al.Clinical characteristics of refractory COVID-19 pneumonia in Wuhan, China. Clin Infect Dis. 2020; ciaa270. Available from: 10.1093/cid/ciaa270.

[74] Pan F, Ye T, Sun P, Gui S, Liang B, Li L, et al.Time course of lung changes on chest CT during recovery from 2019 novel coronavirus (COVID-19) pneumonia. Radiology. 2020:200370. Radiological Society of North America. Available from: https://pubs.rsna.org/doi/10.1148/radiol.2020200370.

[75] Qi D, Yan X, Tang X, Peng J, Yu Q, Feng L, et al.Epidemiological and clinical features of 2019-nCoV acute respiratory disease cases in Chongqing municipality, China: a retrospective, descriptive, multiple-center study. medRxiv. 2020. Available from: http://medrxiv.org/content/early/2020/03/03/2020.03.01.20029397.abstract.

[76] Tang X, Du R, Wang R, Cao T, Guan L, Yang C, et al.Comparison of hospitalized patients with ARDS caused by COVID-19 and H1N1. Chest. 2020. Available from: https://linkinghub.elsevier.com/retrieve/pii/S0012369220305584. 10.1016/j.chest.2020.03.032 PMC7151343 32224074

[77] Tian S, Chang Z, Wang Y, Wu M, Zhang W, Zhou G, et al.Clinical characteristics and reasons of different duration from onset to release from quarantine for patients with COVID-19 outside Hubei province, China. medRxiv. 2020. Available from: http://medrxiv.org/content/early/2020/03/23/2020.03.21.20038778.abstract. 10.3389/fmed.2020.00210 PMC7235406 32574322

[78] Tian S, Zhu X, Sun X, Wang J, Zhou Q, Wang C, et al.Longitudinal analysis of laboratory findings during the process of recovery for patients with COVID-19. medRxiv. 2020. Available from: http://medrxiv.org/content/early/2020/04/07/2020.04.04.20053280.abstract.

[79] Wu J, Liu J, Zhao X, Liu C, Wang W, Wang D, et al.Clinical characteristics of imported cases of coronavirus disease 2019 (COVID-19) in Jiangsu Province: a multicenter descriptive study. Clin Infect Dis. 2020. Available from: https://academic.oup.com/cid/article/doi/10.1093/cid/ciaa199/5766408. 10.1093/cid/ciaa199 PMC7108195 32109279

[80] Wu C, Chen X, Cai Y, Xia J, Zhou X, Xu S, et al.Risk factors associated with acute respiratory distress syndrome and death in patients with coronavirus disease 2019 pneumonia in Wuhan, China. JAMA Intern Med. 2020. Available from: https://jamanetwork.com/journals/jamainternalmedicine/fullarticle/2763184. 10.1001/jamainternmed.2020.0994 PMC7070509 32167524

[81] Wu F, Wang A, Liu M, Wang Q, Chen J, Xia S, et al.Neutralizing antibody responses to SARS-CoV-2 in a COVID-19 recovered patient cohort and their implications. medRxiv. 2020. Available from: https://www.medrxiv.org/content/10.1101/2020.03.30.20047365v2.

[82] Xiao G, Hu H, Wu F, Sha T, Huang Q, Li H, et al.Acute kidney injury in patients hospitalized with COVID-19 in Wuhan, China: a single-center retrospective observational study. medRxiv. 2020. Available from: http://medrxiv.org/content/early/2020/04/08/2020.04.06.20055194.abstract. 10.12122/j.issn.1673-4254.2021.02.01 PMC7905239 33624587

[83] Xie H, Zhao J, Lian N, Lin S, Xie Q, Zhuo H. Clinical characteristics of non-ICU hospitalized patients with coronavirus disease 2019 and liver injury: a retrospective study. Liver Int. 2020. Available from: https://onlinelibrary.wiley.com/doi/abs/10.1111/liv.14449. 10.1111/liv.14449 PMC7228333 32239591

[84] Xu S, Fu L, Fei J, Xiang HX, Xiang Y, Tan ZX, et al.Acute kidney injury at early stage as a negative prognostic indicator of patients with COVID-19: a hospital-based retrospective analysis. medRxiv. 2020. Available from: http://medrxiv.org/content/early/2020/03/26/2020.03.24.20042408.abstract.

[85] Yan D, Liu XY, Zhu YN, Huang L, Dan BT, Zhang GJ, et al.Factors associated with prolonged viral shedding and impact of Lopinavir/Ritonavir treatment in patients with SARS-CoV-2 infection. medRxiv. 2020. Available from: http://medrxiv.org/content/early/2020/03/30/2020.03.22.20040832.abstract. 10.1183/13993003.00799-2020 PMC7241115 32430428

[86] Yuan J, Zou R, Zeng L, Kou S, Lan J, Li X, et al.The correlation between viral clearance and biochemical outcomes of 94 COVID-19 infected discharged patients. Inflamm Res. 2020. Available from: 10.1007/s00011-020-01342-0. 10.1007/s00011-020-01342-0 PMC7103893 32227274

[87] Zeng Z, Sha T, Zhang Y, Wu F, Hu H, Li H, et al.Hypertension in patients hospitalized with COVID-19 in Wuhan, China: a single-center retrospective observational study. medRxiv. 2020. Available from: http://medrxiv.org/content/early/2020/04/11/2020.04.06.20054825.abstract.

[88] Zhang G, Hu C, Luo L, Fang F, Chen Y, Li J, et al.Clinical features and outcomes of 221 patients with COVID-19 in Wuhan, China. medRxiv. 2020. Available from: http://medrxiv.org/content/early/2020/03/06/2020.03.02.20030452.abstract. 10.1016/j.jcv.2020.104364 PMC7194884 32311650

[89] Zhao W, Yu S, Zha X, Wang N, Pang Q, Li T, et al.Clinical characteristics and durations of hospitalized patients with COVID-19 in Beijing: a retrospective cohort study. medRxiv. 2020. Available from: http://medrxiv.org/content/early/2020/03/30/2020.03.13.20035436.abstract.

