# Supplementary material for: COVID-19 length of hospital stay: a systematic review and data synthesis
Source: BMC Med. 2020 Sep 3;18:270. doi: 10.1186/s12916-020-01726-3 (PMC7467845; doi:10.1186/s12916-020-01726-3)
Supplement: Supplementary file 1 — Additional file 1 List of search terms. Supplementary Table A. Description of information extracted from included studies. Fig SA: LoS by disease severity. Fig SB: LoS by study median age. Fig SC: LoS by outcome status. Fig SD: Summary distributions for China/other and total/ICU. Fig SE: Sensitivity of length of stay (LoS) with and without overlapping populations removed. Fig SF: Sensitivity analysis of summary distributions to weighting. [file 12916_2020_1726_MOESM1_ESM.pdf]

COVID-19 length of hospital stay: a systematic review and data synthesis

Supplementary material

Search terms used for Embase and Medline:

1. 2019-nCov.mp.
2. SARS-CoV2.mp.
3. COVID-19.mp.
4. coronavirus.mp.
5. exp Coronavirus Infections/
6. exp Coronavirus/
7. exp Severe Acute Respiratory Syndrome/
8. exp "Length of Stay"/
9. length of stay.mp.
10. Hospital duration.mp.
11. Admission duration.mp.
12. Admission length.mp.
13. hospital*.mp.
14. 1 or 2 or 3 or 4 or 5 or 6 or 7
15. 8 or 9 or 10 or 11 or 12 or 13
16. limit 15 to yr="2020 -Current"
17. 14 and 15 and 16
18. remove duplicates from 17

Search terms used for Medrxiv:

"(Coronavirus OR Covid-19 OR 2019-nCov OR SARS-CoV-2) AND (Length of stay OR Admission duration OR Admission Length OR Hospital)" and posted between "01 Jan, 2020 and 12 Apr, 2020"

Supplementary Table A. Description of information extracted from included studies.

**Variable**		**Description**
General study characteristics	Author	First author of the study
	Title	Title of the paper or pre-print
	Publication date	Publication date or pre-print date
	Status	Publication status (published or pre-print)
	Study dates	This is either related to the admission or discharge dates, depending on the study
	Country or countries	Country or countries from which the data was collected
	Region or province	Specific region within the country from which the data was collected
	Study population	Study population included within the study, including which hospitals patients were recruited from
	Study design	Type of study
LOS sample characteristics	Population size	Size of the population for which LOS was reported
	Age	Median or mean age of the population for which LOS was reported
	Sex	Sex (% male) of the population for which LOS was reported

	LOS	Length of stay, broken down by ICU admission and general admission when reported, and by sample characteristics reported (for example, disease severity, comorbidities and treatment groups).
	LOS endpoint	LOS endpoint - either death or discharge. If ICU LOS, transfer to another ward was also included.
	LOS oxygen support	LOS for invasive and non-invasive mechanical ventilation and oxygen support
	Follow-up complete	Follow-up was considered complete if all patients had a discharge outcome (death or discharge) at the end of the study. If patients remained in hospital at the end of the study period follow-up was considered incomplete with regards to LOS.
	LOS symptom onset	Symptom onset to admission, discharge or death
	Notes	Issues and comments on the study

Supplementary Table B: Database of all studies included within the review. *Available as an excel spreadsheet.*

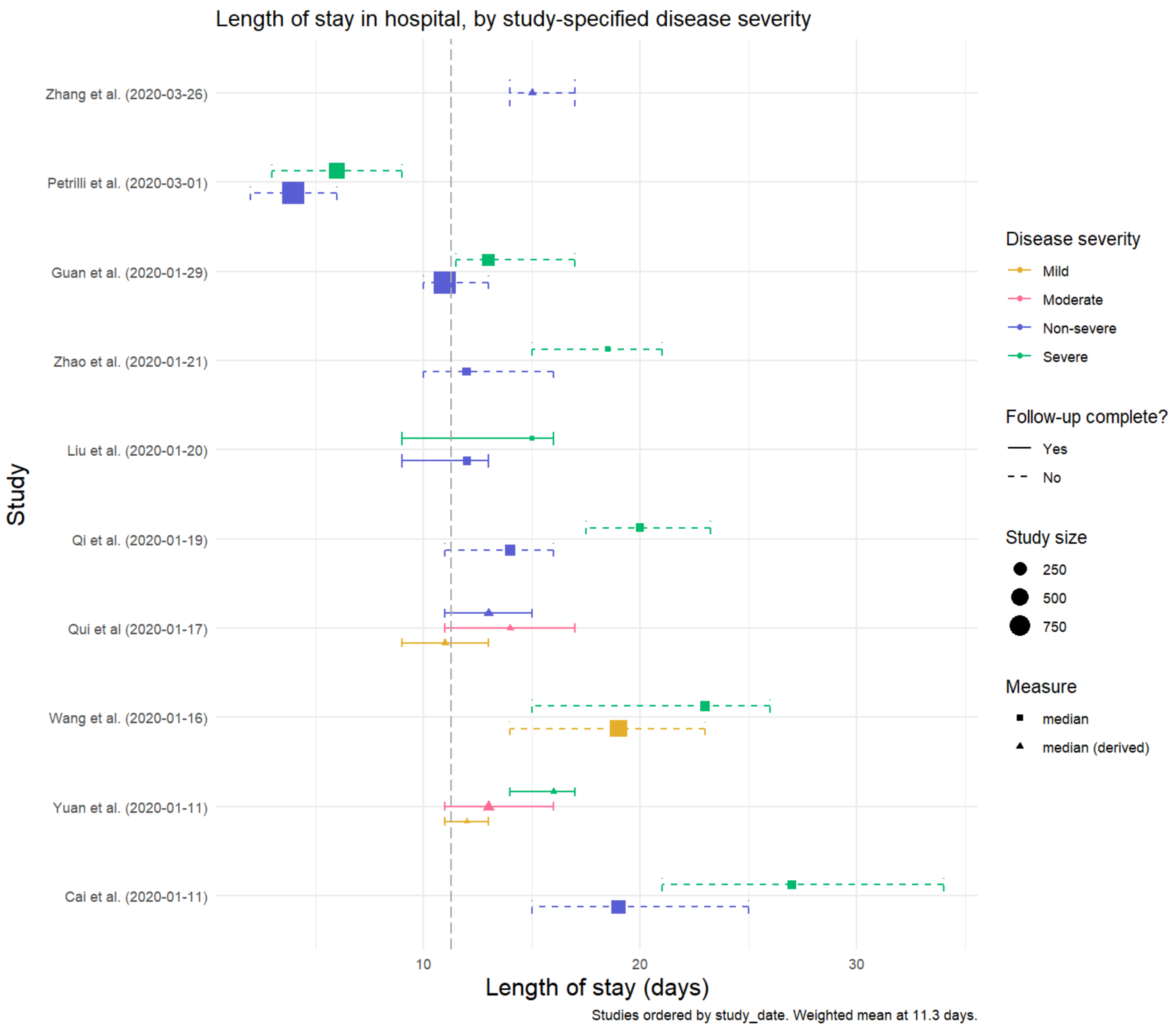

Supplementary figure A: Length of stay (general or ICU) by disease severity, as defined within each study. Note that, amongst those studies which reported LOS estimates for specific severity groups, the definitions applied to stratify participants are inconsistent and as such the subgroup estimated cannot be directly compared between studies. Within each study, however, there does appear to be a trend towards longer stays for more severe cases. None of these studies report a specific estimate for non-survivors, who could be defined as the most severe cases and may have had shorter stays.

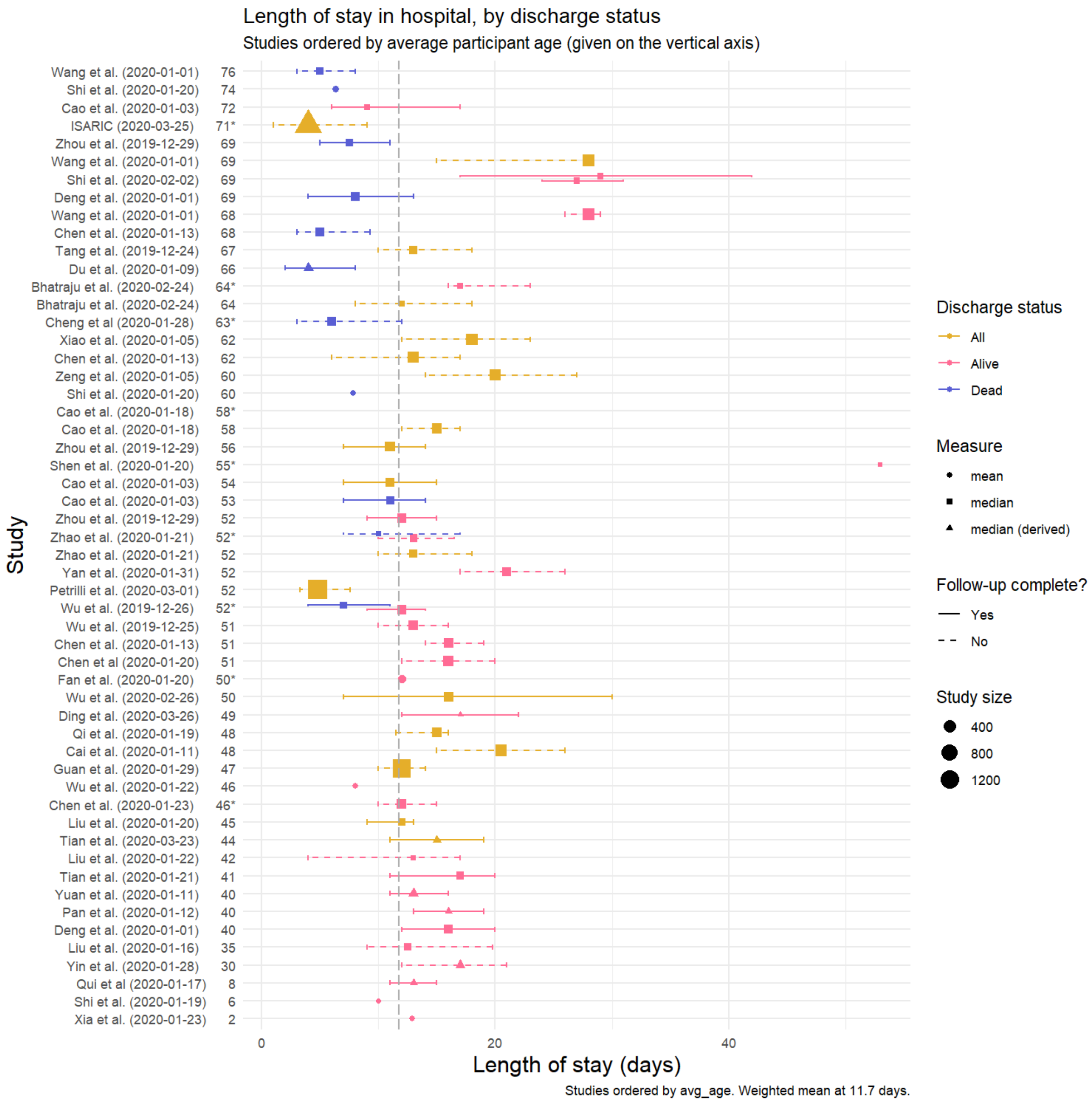

Supplementary figure B: Total length of stay, by discharge status and ordered by average patient age. "Average" age represents a median or mean depending on what was reported. In some cases, age distribution was only reported for the overall study cohort, and not for the specific subgroup within which LOS was reported (i.e. those with observed discharge). These cases are highlighted with an asterisk (*).

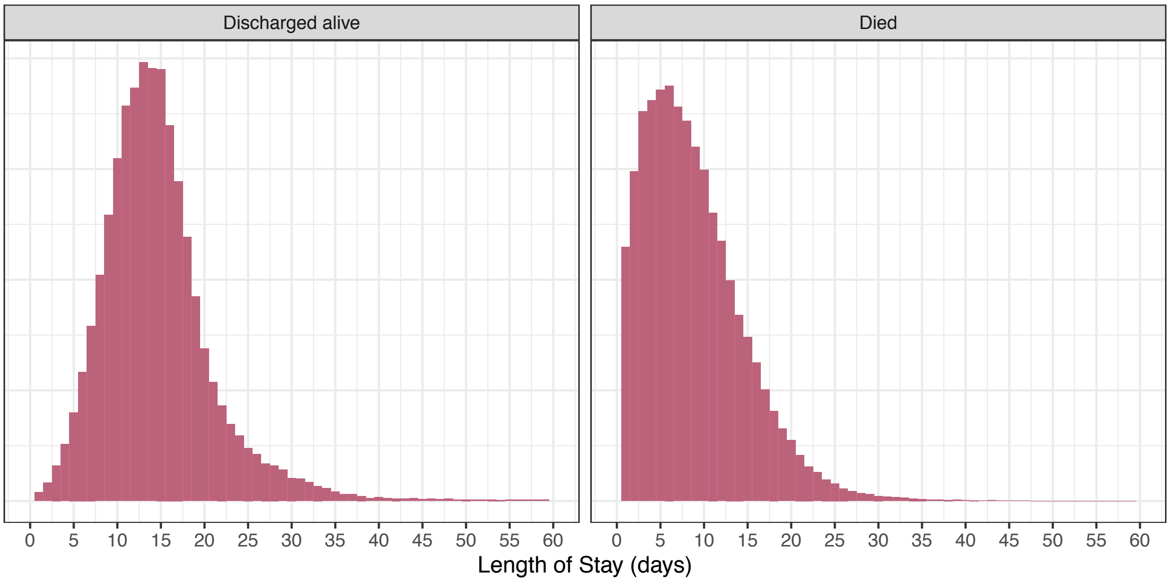

Supplementary Figure C: Total length of stay (LoS) for all studies which reported total LoS for patients discharged alive (n = 10) and patients who died (n = 5). Samples from the overall LOS distributions where follow-up was complete for all study participants. For each subset, 100000 draws were taken.

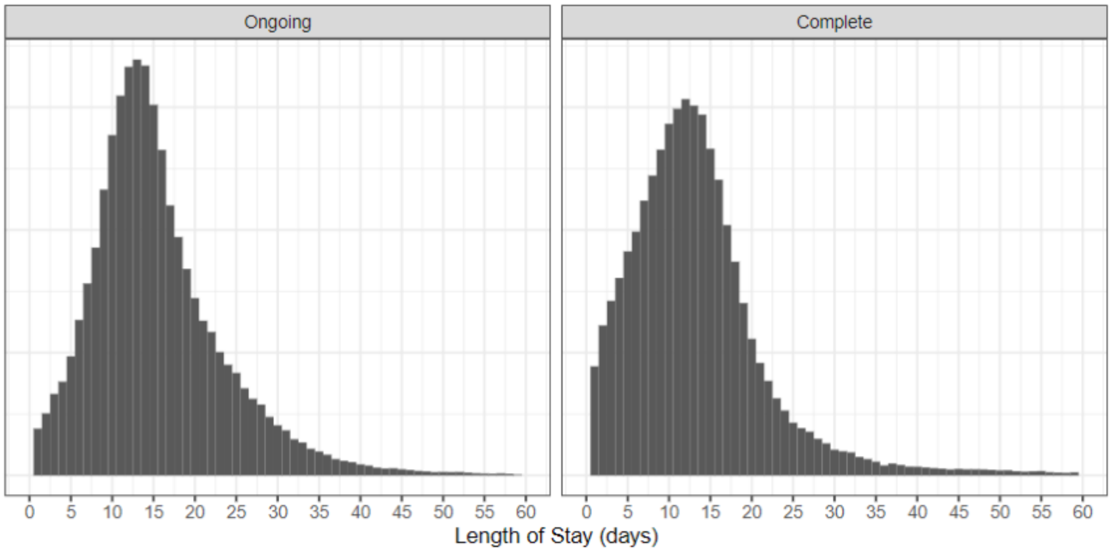

Supplementary Figure D: Total length of stay (LoS) for studies from China which reported completed (n = 22) and incomplete follow-up (n = 15) with respect to LoS. Samples from the overall LOS distributions. For each subset, 100000 draws were taken.

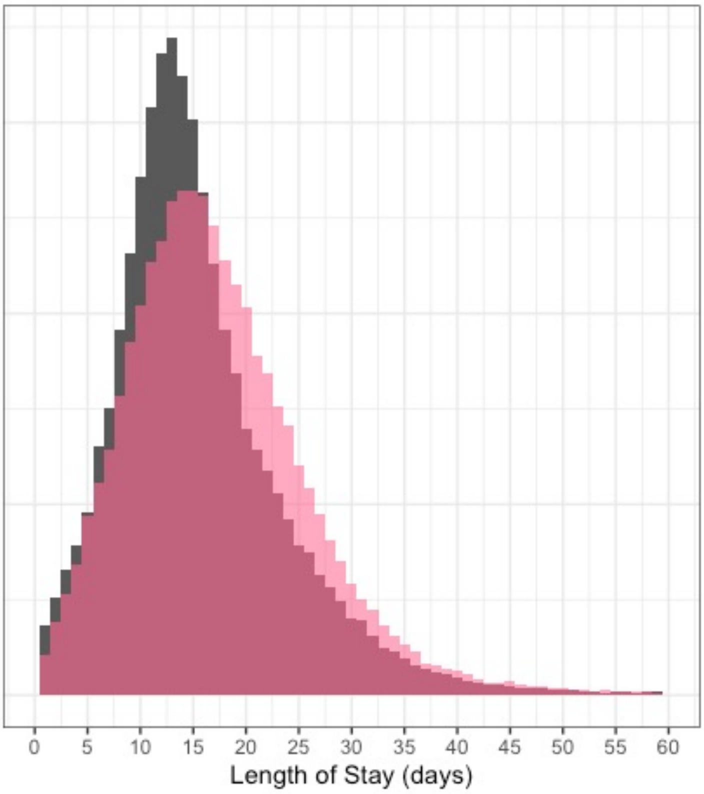

Supplementary Figure E. Sensitivity of length of stay (LoS) with and without overlapping populations removed. Original distribution from studies reporting total LoS in China (grey) is shown with the distribution where overlapping study populations have been removed (overlaid, red).

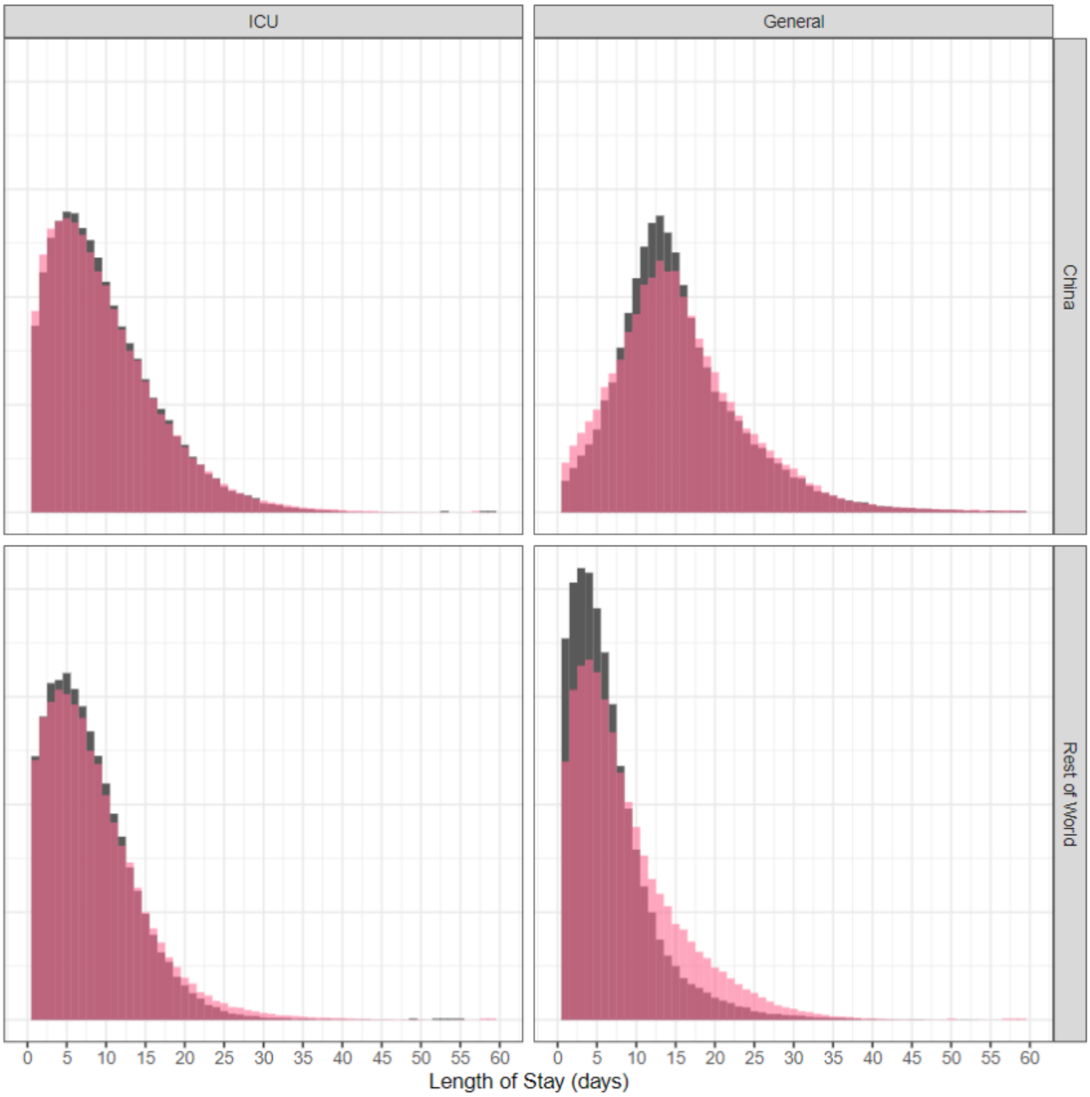

Supplementary Figure F: Sensitivity of combined LOS distributions to weighting. Original distributions (grey) are shown with unweighted versions overlaid (red), in which all studies were weighted equally.
